# Arbuscular mycorrhizal fungi in soil, roots and rhizosphere of *Medicago truncatula*: diversity and heterogeneity under semi-arid conditions

**DOI:** 10.7717/peerj.6401

**Published:** 2019-03-01

**Authors:** Neji Mahmoudi, Cristina Cruz, Mosbah Mahdhi, Mohamed Mars, Maria F. Caeiro

**Affiliations:** 1 Faculté des Sciences de Gabès, Unité de recherche, Biodiversité et Valorisation des Bio-ressources en Zones Arides (BVBZA), Erriadh Zrig, Tunisie; 2 Faculdade de Ciências da Universidade de Lisboa, Centre for Ecology, Evolution and Environmental Changes (cE3c), Lisboa, Portugal; 3 Center for Environmental Research and Studies, Jazan University, Jazan, Kingdom of Saudi Arabia; 4 Faculdade de Ciências da Universidade de Lisboa, Centro de Estudos do Ambiente e do Mar (CESAM), Lisboa, Portugal

**Keywords:** Pyrosequencing, Microbial diversity, AMF, Soil heterogeneity, Heterogeneity

## Abstract

Mycorrhizal symbioses are considered indicators of ecosystem biodiversity. However, their diversity and relevance in arid and semi-arid ecosystems are poorly understood. This study addressed this subject, the main objective being to evaluate arbuscular mycorrhizal fungi (AMF) diversity and heterogeneity in a semi-arid region. Samples of bulk and rhizosphere soil and fine roots of *Medicago truncatula* were collected at four different sites with the same aridity index (6.1), in Bou-Hedma National Park, Tunisia, a semi-arid ecosystem. AMF taxa were assessed by 454- pyrosequencing and identified by BLAST matching of operational taxonomic units (OTUs) against the Maarj*AM* database, targeting AMF SSU *rRNA* gene diversity. Roots were the hotspots of AMF diversity (107 OTUs out of a total of 138). Of the 138 OTUs, 113 found correspondence in the Maarj*AM* database, with 32 AMF virtual taxa (VTX),****19 Site-exclusive (SE) and 13 common to at least two sites (Non-site exclusive, NSE); the remaining 25 OTUs grouped in 16 putative new AMF taxa (pNTX), each one consisting of OTUs sharing pairwise distances not higher than 3%. We found a high diversity and heterogeneity of AMF across the four sites, which showed, in a regression analysis, significant relation to six out of the eight environmental parameters evaluated: grazing activity and soil texture, electrical conductivity, organic matter, total phosphorus and total nitrogen. AMF colonization of plants also presented significant differences among the four sites, as well as spore density, microbial biomass and several enzymatic activities (dehydrogenase, β-glucosidase and phosphatase) evaluated in rhizosphere soils. The four sites clustered in two groups in a hierarchical clustering evaluation based on their AMF diversity (total numbers of OTU, VTX and pNTX) and the parameters referred above. The crucial role of abiotic factors, other than aridity index, on AMF community composition, was evidenced by the high heterogeneity found between AMF communities across sites under identical aridity conditions.

## Introduction

Semi-arid ecosystems cover 38 to 41% of all ecosystems (Reynolds et al., 2007). They have been subjected to accelerated desertification mainly due to pressures associated with anthropogenic impacts (i.e., grazing) and climate changes, which caused the decline of forests, regression and extinction of many pastoral and forage species, accelerated soil degradation and changes in soil microbial communities (Fterich, Mahdhi & Mars, 2012; Abdallah & Chaieb, 2013). It is thus important to understand the factors determining the response of vegetation and soil communities to climate changes in order to develop adequate means of conservation. While plant community changes are better studied (Hodgson et al., 2005), we are just about to understand the impact of climate changes on soil organisms and their role on semi-arid ecosystems (Delgado-Baquerizo et al., 2016). Direct interaction of soil organisms with plants, such as arbuscular mycorrhizal fungi (AMF), might be of particular relevance.

AMF belong to the phylum Glomeromycota (Schüßler, Schwarzott & Walker, 2001) and are obligatory plant symbionts. More than 80% of the terrestrial plant species form symbiotic associations with AMF communities, which play a key role in plant performance (Munkvold et al., 2004; Smith & Read, 2008) at individual and ecosystem levels. AMF are important regulators of ecosystems dynamics and functionality, enhancing phosphorus acquisition and soil aggregation, structure and fertility (van der Heijden, Bardgett & van Straalen, 2008). They also may promote plant growth and protect their hosts from pathogens (Pozo & Azcon-Aguilar, 2007), increasing their tolerance to abiotic and biotic stresses (Smith & Read, 2008) and contributing to improve plant fitness. In fragile ecosystems, AMF are crucial for the survival of plant species, by allowing their access to the limited soil resources (Chaudhary et al., 2014), thus being of major importance in arid and semi-arid ecosystems (Zhao et al., 2017). Due to their ability in establishing symbiotic associations with most terrestrial plants, AMF can be used to maintain the stability of semi-arid environments and to preserve ecosystems from the aridification and desertification related to climate changes (high temperatures, low rainfall and long dry seasons (Barea et al., 2011; Neuenkamp et al., 2018).

Generally, the symbiotic interactions between AMF and plant, are considered to be non-specific (Smith & Read, 2008): the same plant is colonized by different AMF species, which may colonize different plant species and create networks of communication and channels for solute transport between distinct plants at variable distances (Gollotte, Van Tuinen & Atkinson, 2004). Some AMF have more influence on nutrient use efficiency, others on plant development and others on plant defense (Smith & Read, 2008). The final outcomes of the mycorrhization depend on the AMF and plant species involved in the symbioses (Munkvold et al., 2004; Jansa, Smith & Smith, 2008). It was documented that diverse AMF communities affect positively the diversity and productivity of natural ecosystems (van der Heijden, Bardgett & van Straalen, 2008). It was also reported that plant growth and development are improved by interaction with more diverse AMF taxa (van der Heijden et al., 1998; Wagg et al., 2011), which results in lower levels of plant stress, due to complementarity effects within the AMF communities, in particular regarding the acquisition of the limited soil resources typical of semi-arid ecosystems.

Soil physical, chemical and biological properties are important determinants of soil and plant AMF community composition (Hallett et al., 2009; Gai et al., 2009; Oehl et al., 2010). Soil disturbance associated with ecosystem and agro-system management tend to have a positive effect on AMF diversity if associated with increased soil organic matter accumulation, soil aggregates and microbial organic carbon (Smith & Read, 2008; Oehl et al., 2010). If disturbance is associated with decreasing soil organic matter, the usual result is a decrease in AMF diversity (Toljander et al., 2008). Moreover, most of the time, the intensity of the management, and not the type of management per se, has the biggest effect on AMF diversity. For instance, grazing may have very distinct effects on AMF communities, depending on the initial characteristics of the system, the grazing species, and the number of grazers per unit area (Mendoza et al., 2011). On the other end, global change threats, aridity in particular, tend to decrease AMF diversity and abundance by decreasing soil carbon and nitrogen storage due to primary production constraints (Delgado-Baquerizo et al., 2016).

To understand the effects of mycorrhization on plant development and its impact on the ecology of plant communities (i.e., adaptation of plants to their environment, distribution of plants in space, survival strategies, symbiotic associations...), it is important to assess the composition of AMF communities in the rhizosphere (Antunes et al., 2011). Traditionally, the identification of AMF species was based on few morphological characters (Morton & Benny, 1990), which has a high potential for misidentification (Redecker & Raab, 2006). DNA-based identification is an alternative approach (Gorzelak et al., 2012), which may be used in combination with AMF morphological identification. Previous works have reported the composition of AMF communities in different ecosystems (i.e., Öpik et al., 2006; Wang et al., 2015; Alguacil et al., 2016); however, few studies have emphasized the role of AMF in sustaining plant cover in semi-arid and arid ecosystems (Varela-Cervero et al., 2015; Alguacil et al., 2016; Zhao et al., 2017).

*Medicago truncatula* (barrelclover) grows naturally across a broad range of stressful edaphic environments throughout the Mediterranean ecosystem (Maren et al., 2010) and can form nodules with nitrogen-fixing bacteria (rhizobia) and endomycorrhiza with AMF (Chen et al., 2009; Gomes et al., 2015). It is the consensus that the broad distribution of *M. truncatula* is not only due to its characteristics, but also to the symbioses the plant establishes with distinctive microbial communities (rhizobia and AMF) supporting its growth under stressful conditions (i.e., drought, salinity...) (Maren et al., 2010). Despite the wide distribution of *M. truncatula* along environmental gradients in semi-arid ecosystems, only few studies are available about AMF community patterns associated with this host plant.

The aim of this study, using high-throughput sequencing, was to assess the diversity and composition of AMF communities in response to variation of environmental conditions (mainly soil properties and resources availability) in different semi-arid environments with a similar aridity index. To address this objective, we assessed the AMF diversity in four sites showing distinct properties (soil features, vegetation, altitude, management practices) while sharing the same aridity index and the presence of a plant species—*M. truncatula*—with recognized high levels of AMF colonization (Sonja et al., 2003; Andrea et al., 2005). Three belowground compartments were evaluated at each site: bulk soil, *M. truncatula* rhizosphere soil and *M. truncatula* roots.

## Materials and Methods

### Study area and sampling

**Figure 1:**
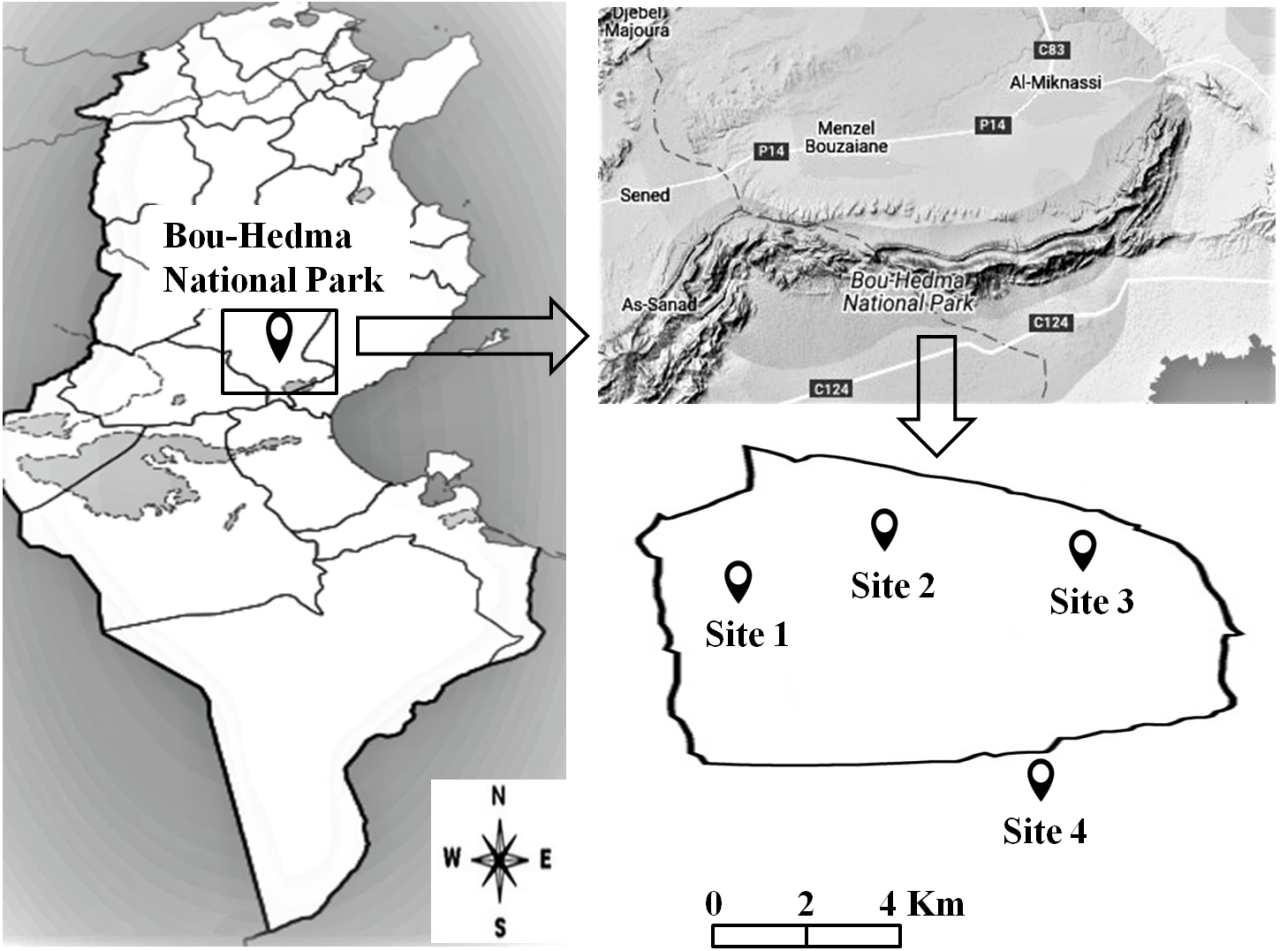
Location of the studied sites inside and outside the Bou-Hedma National Park.

An area at Bou-Hedma National Park, situated in the Governorate of Sidi Bouzid, in central-southern Tunisia (Fig. 1), was studied under the Permit 1043 from the Forestry Service of the Minister of Agriculture of Tunisia Republic. Four sites (three inside the Park and one outside) were selected, differing in altitude, vegetation, grazing intensity and soil texture, physical and chemical properties. Site 1 was an open area near an *Acacia* (*Acacia tortilis* subsp. *raddiana*) population; Site 2 was located near a seasonal water course and Site 3 on a mountain summit (600 m altitude). These three sites were subjected to light grazing (1 animal per 40 ha) by Saharan antipodeans (*Addax nasomaculatus* and *Oryx leucoryx*) and some ostriches (*Struthio camelus*). Site 4, the one outside the Park, was subjected to intensive grazing by herds of domesticated sheep, goats and camels (80 animals per 40 ha) (Fterich, Mahdhi & Mars, 2012; Abdallah & Chaieb, 2013), as well as to management practices (conventional tillage; conventional and organic farming under irrigation or non-irrigation conditions). The four sites presented the same aridity index (6.1), calculated according to De Martonne (1926) using the equation: AI = P/(T+10), where *P* (mm) is the annual precipitation and T (°C) the annual mean temperature.

From each studied site, three replicate samples were collected from each one of three compartments: roots of the dominant plant species *M. truncatula* (to a depth of 10–20 cm), rhizosphere soils of the sampled plants and bulk soil (4 × 3 × 3 = 36 samples). Roots were carefully collected in order to access the fine active roots where AMF colonization occurs. Rhizosphere soil was obtained by gently digging below and around the root zone of each root replicate. Bulk soil samples were collected by digging at a depth of 10–20 cm, at least 10 m away from each sampled plant.

Roots were washed with tap water either for the evaluation of the AMF colonization status or to be cut in pieces stored in microtubes at −20 °C, until DNA extraction. Soil samples were stored at 4 °C for a few days until soil analyses and DNA extraction.

### Soil analysis

The bulk soil and rhizosphere soil samples (4 sites × 3 replicates = 12 samples for each compartment) were subjected to the following analyses.

Soil pH was measured in soil water suspensions (10 g of soil/100 mL of water), with a selective electrode.

Electrical conductivity was determined using a conductivity meter (AFNOR, 1987).

Total soil organic carbon was determined using the Walkley & Black (1934) method.

Soil texture was calculated according to Robinson’s pipette method (Naanaa & Susini, 1988). Organic matter was evaluated indirectly, starting from the determination of the organic carbon content of soil.

Total phosphorus and nitrogen were determined by acid degradation reaction (first step) followed by a dosing step using an automated system.

The carbon of the microbial biomass (Cmic) was determined by the “fumigation–extraction” method (Amato & Ladd, 1988) which consists of using ninhydrin-N reactive compounds extracted from soils (three replicates of 20 g) with KCl after a 10-day fumigation period.

Soil respiration (C-CO_2_ released) was determined according to Öhlinger (1995).

The metabolic quotient (qCO_2_) was calculated by dividing the C-CO_2_ released by the microbial biomass carbon (Cmic) content.

Phosphatase and β-glucosidase activities were measured according to Caravaca et al. (2005) through the absorbance at 398 nm to evaluate the p-nitrophenol (PNP) formed. Dehydrogenase activity was measured as described by Garcia, Hernandez & Costa (1997) through the absorbance at 490 nm after a 20 h incubation period in the dark, to determine the iodonitrotetrazolium formazan (INTF) formed.

All assays were performed in triplicate.

### AMF colonization status

AMF colonization was evaluated by observation of 30 root fragments per plant (a total of 4 sites × 3 replicates × 30 fragments = 360 root fragments), following the method of Phillips & Hayman (1970). Briefly, root segments (1–2 cm) were submerged in 10% KOH at 90 °C for 45 min, bleached in H_2_O_2_ for 3 min and acidified with 1% HCl. Then, the root segments were stained for 90 min at 60 °C in 0.05% trypan blue. Frequencies of AMF arbuscules and vesicles were calculated according to Giovannetti & Mosse (1980) as follows: Mycorrhizal Frequency (F%) = 100 ×  (N-NO)/N, where N represents the number of observed fragments, and NO the number of non-mycorrhizal fragments. The levels of mycelium inside the roots were determined by assigning an index of mycorrhization from 0 to 5; the Mycorrhizal Intensity (M%), defined as the percentage of roots colonized by AMF (Derkowska et al., 2008), was calculated as: M% = (95*n*5 + 70*n*4 + 30*n*3 + 5*n*2 + *n*1)∕*N*, where *n* = number of fragments assigned with the index 0, 1, 2, 3, 4 or 5 of colonization (0- no, 1- trace, 2- less than 10%, 3–11 to 50%, 4–51 to 90%, 5- more than 91%).

AMF spores occurring in rhizosphere and bulk soil samples were extracted following the wet sieving method described by Gerdemann & Nicolson (1963). Composite soil samples of 100 g were sieved through three nested sieves with mashes of 1,000, 100 and 32 µm. Retrieved AMF spores placed in Petri dishes were counted under a stereomicroscope (40×  magnification) and average numbers were calculated per 100 g of dry soil.

### DNA extraction and amplification

Individual samples from three plant roots (0.6 g of fresh root material/sample) and from three rhizosphere and three bulk soils (1 g of soil/sample) from each site (a total of 36 samples) were used for DNA extractions carried out with either the EZ-10 Spin Column Plant Genomic DNA Miniprep Kit or the EZ-10 Spin Column Soil DNA Miniprep Kit, according to the recommendations of the manufacturer (Bio-Basic, Canada). The extracted DNA was eluted in 20 µl of Elution buffer.

Partial small subunit (SSU) of the nuclear ribosomal RNA (*rRNA*) gene fragments were amplified using a nested PCR protocol with the universal eukaryotic primers NS1and NS4 in the first PCR and the specific AM fungal primers NS31 and AM1 in the second (nested) PCR (Table 1). Both PCR amplifications were carried out in a T personal1 cycler (Biometra, Göttingen, Germany) with reaction mixtures of 10 µl using the Hot Star Taq DNA polymerase (Qiagen, Hilden, Germany), Qiagen buffer, 0.5 mM of each primer and 2 µl of DNA (either plant roots, rhizosphere and bulk soil DNA samples in the first PCR or the first PCR product, in the nested PCR).The PCR conditions were, 95 °C for 10 min followed by 30 cycles at 94 °C for 45 s, 45 °C for 45 s and 72 °C for 90 s, followed by a final extension period of 3 min at 72 °C. Negative controls (sterile water) were used in all PCR reactions. Size and yields of the amplification products were estimated by electrophoresis using 1% agarose gels in TBE (Tris/Borate/EDTA) buffer containing ethidium bromide. PCR products were purified using the DNA Clean & Concentrator kit (Zymo Research, Irvine, CA, USA) according to the recommendations of the manufacturer. After quantification using a NanoDrop™ Spectrophotometer ND-1000 (Thermo Fisher Scientific, Wilmington, DE, USA), the three amplification products from each type of sample were pooled at equimolar concentrations to obtain one composite DNA sample of each type of compartment (roots/ rhizosphere soils/ bulk soils) for each site, i.e., a total of 12 samples.

**Table 1:** PCR primers used in this study.

Primer	Nucleotide sequence (5′to 3′)	Target organism	Reference
NS1	GTA GTC ATA TGC TTG TCT C	Eukaryota	White et al. (1990)
NS4	CTT CCG TCA ATT CCT TTA AG	Eukaryota	White et al. (1990)
NS31	TTG GAG GGC AAG TCT GGT GCC	Glomeromycota	Simon, Lalonde & Bruns (1992)
AM1	GTT TCC CGT AAG GCG CCG AA	Glomeromycota	Helgason et al. (1998)

### Pyrosequencing

DNA samples were amplified for the hypervariable AM region with fusion primers containing the Roche-454 A and B Titanium sequencing adapters, an eight-base barcode sequence in fusion primer A and AM1 and NS31 primers, respectively in fusion primers A and B. PCR reactions were performed for each sample using 1×  Advantage SA PCR Buffer, 0.2 µM of each PCR primer (fusion primers A and B), 0.2 mM dNTPs (Bioron, Ludwigshafen am Rhein, Germany), 5% DMSO (Molecular Probes, Life Technologies, Carlsbad, CA, USA), 1×  Advantage 2 Polymerase Mix (Clontech, Mountain View, CA, USA), and 1µl of 1:10 diluted PCR product in a total volume of 40 µl. The PCR conditions involved a 4 min denaturation at 94 °C, followed by 15 cycles of 94 °C for 30 s, 61 °C for 45 s and 68 °C for 60s and a final extension at 68 °C for 10 min. Negative controls were included for all amplification reactions. Electrophoresis of the PCR products was undertaken on a 1% (w/v) agarose gel and the ∼600 bp amplified fragments were purified using AMPure XP beads (Agencourt, Beckman Coulter, Brea, CA, USA) according to manufacturer’s instructions. The amplicons were quantified by fluorimetry with PicoGreen dsDNA quantitation kit (Invitrogen, Life Technologies, Carlsbad, CA, USA), pooled at equimolar concentrations and sequenced in the A direction with GS 454 FLX Titanium chemistry, according to manufacturer’s instructions (Roche, 454 Life Sciences, Branford, CT, USA) at Genoinseq (Cantanhede, Portugal).

### Bioinformatics data analysis

The raw pyrosequencing reads were processed at GenoInSeq (Cantanhede, Portugal), using an automatic pipeline implemented at this Laboratory. In a first step, sequencing reads were assigned to the appropriate samples based on the respective barcode. Then, reads were quality filtered (Q20) to minimize the effects of random sequencing errors, by elimination of sequence reads with <100 bp and sequences that contained more than two undetermined nucleotides (N). Sequences were additionally cut for the reverse primer, if present. Finally sequences with more than 50% of low complexity regions, determined by DustMasker (Sogin et al., 2006) and chimera sequences, identified by UChime (Edgar et al., 2011), were discarded. The sequences were grouped by USearch (Edgar, 2010) according to a phylogenetic distance of 3%, creating the Operational Taxonomic Units (OTU). Rarefaction curves and Chao1 indices were calculated using the Mothur software (Schloss et al., 2009). The taxonomy of each OTU was identified through a BLAST search against the nt@ncbi. The best hits were selected and subjected to further quality control. All sequences with an alignment of more than 40% as well as those with an *E*-value lower than 1e−were accepted. Additionally, a bootstrap test was applied to the OTUs to assess the correct *E*-value scores and identify the least common taxonomy level. Only the sequences with a bootstrap greater than 70% after 100 replicates, as obtained by seqBoot from Phylip package (Felsenstein, 1989), were kept. The taxonomic assignment of the OTUs was completed with the attribution of the NCBI taxonomy identification number, which allowed the complete taxonomy construction of all identified organisms. “Unidentified” was a designation applied in the present study, for identifications done only to domain or to kingdom levels.

The raw sequence reads were deposited at NCBI, in the SRA database with the following accession: SRP153928.

### Glomeromycota identification

Each Glomeromycota OTU was blasted against SSU sequences from the Maarj *AM* database (http://maarjam.botany.ut.ee/) (Öpik et al., 2010) and was identified based on the virtual taxon (VTX) with which it shares the highest identity (ID) value, for ID ≥ 97%. All identifications were based on coverage (CV) values higher than 75%. When identical ID values allowed the attribution to more than one VTX, the identification was based on the highest coverage value. When no match was found or for ID<97%, a VTX number was not attributed and the OTU was considered a putative new taxon (pNTX). All OTUs corresponding to putative new taxa (pNTX) were subjected to an additional BLAST search against the nt@ncbi and to a pairwise distance evaluation using the Maximum Composite Likelihood (MCL) approach. Matrices of pairwise distances based on a 277 nt alignment were generated with Mega 7.0 (Kumar, Stecher & Tamura, 2016) and OTUs sharing distances less than 3% where considered as belonging to the same pNTX and specified by an alphabetic letter.

A total of 138 sequences were submitted to GenBank (File SUB163348: MG321415 –MG321557) (Table S1).

### Statistical analyses

Analyses of variance (ANOVA) using the SAS statistical package were performed to test differences between studied parameters. Least significant difference values at the 5% levels of significance (*P* ≤ 0.05) were calculated.

The Shannon diversity index (H′) was calculated using the (*H*′) =  − ∑ (Pi) ln (Pi), where Pi = ni/N (ni is the number of species i, and N is the total number of species). Pielou’s evenness index (*J*′) was also calculated: *J*′ = *H*∕*H*_max_ = *H*∕*lnS* (*S* is the number of total species in each site). The Simpson Index (D) was calculated using the following equation: D = 1/ ∑(*Pi*)^2^. To compare the AMF communities between sites, Jaccard’s index was calculated with the equation, *J* = *S*_c_∕*S*_a_ + *S*_b_ + *S*_c_, where *S*_a_ and S_b_ are the number of unique species from samples a and b, respectively, and S_c_ is the number of species common to those samples. A generalized linear mixed model (GLMM), with a logit link function and normal distribution, was used to assess the relevance of environmental parameters (soil texture, pH, E.c, Org. m, TN, TP, altitude level and Grazing activity) in predicting the AMF composition and diversity. Logistic regression analysis was performed in R-3.0.2 software (R Core Team, 2013).

A hierarchical cluster analysis performed with SPSS V.23 was used to compare the AMF communities within the different studied sites, based on soil properties.

## Results

### Soil physical and chemical properties

Soil characteristics differed in texture among sites: Sites 1 and 3 had sandy loam textures, Site 2 had sandy and Site 4 loam textures (Table 2).

**Table 2:** Physical and chemical properties of the studied sites.

Site	Coordinates	Altitude (m)	Grazing intensity	Soil properties					
				Texture	pH	E.c (s m^−1^)	Org.m (%)	T.N (ppm)	T.P (ppm)
1	34.48N 9.46E	100–150	Light	Sandy loam	8.0 ± 0.1^b^	2.3 ± 0.3^a^	1.9 ± 0.2^a^	182 ± 23^a^	7 ± 0.1^c^
2	34.49N 9.52E	≤100	Light	Sandy	8.3 ± 0.1^a^	2.0 ± 0.1^b^	1.1 ± 0.3^c^	125 ± 10^c^	8 ± 0.2^b^
3	34.49N 9.59E	600–700	Light	Sandy loam	8.0 ± 0.2^b^	2.3 ± 0.1^a^	1.4 ± 0.1^b^	150 ± 15^b^	5 ± 0.2^d^
4^*^	34.45N 9.58E	100–150	Intensive	Loam	8.1 ± 0.1^b^	1.7 ± 0.2^c^	0.9 ± 0.1^d^	90 ± 10^d^	14 ± 0.2^a^

**Notes.**

* Located outside Bou-Hedma National Park and subjected to management practices.

E.c electrical conductivity Org.m organic matter T.N total nitrogen T.P total phosphorus

a, b, c and d: significant differences (*P* < 0.05); mean and standard error values (*n* = 3).

All soils were alkaline (pH 8.0–8.3) with electrical conductivities ranging from 1.7 to 2.3 s m^−1^. Statistically, soils from Sites 1 and 3 did not differ from each other for electrical conductivity, while concerning pH values only Site 2 was statistically different (Table 2).

The highest percentage of soil organic matter (1.9%) was observed in Site 1, as well as the highest value of total nitrogen. Soil phosphorus content, ranging from 5 to 14 ppm, varied significantly among the studied sites, the highest value being observed in Site 4. Concerning the three soil properties referred above, significant differences were found between the four sites.

### AMF Colonization and spore density

**Figure 2:**
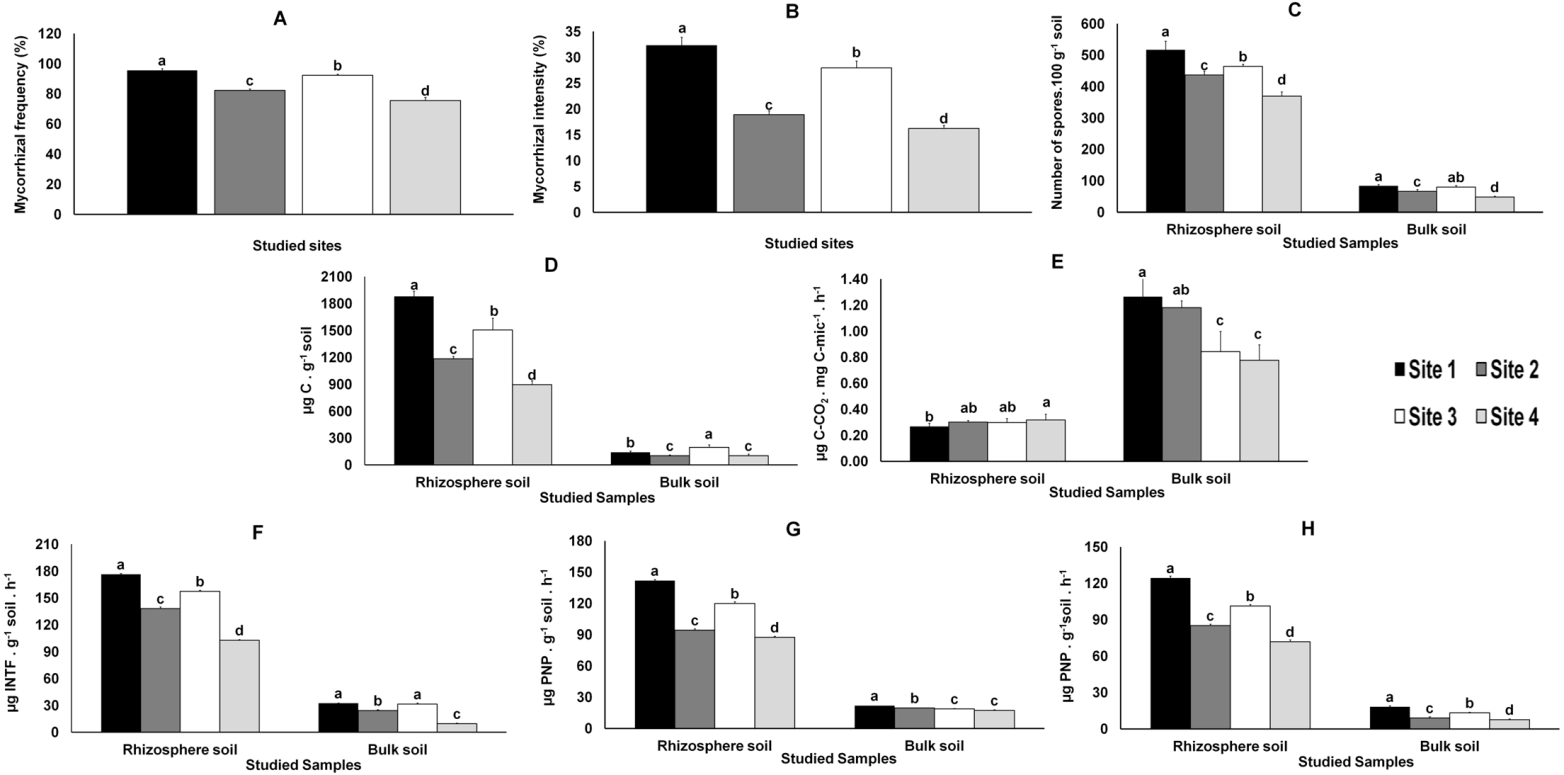
Data from AMF colonization of plant roots and from parameters evaluated in rhizosphere and bulk soils. Data from AMF colonization (mycorrhizal frequency and mycorrhizal intensity) of plant roots (A and B, respectively) and from parameters evaluated in rhizosphere and bulk soil samples from the studied sites: spore density (C), microbial biomass (D), metabolic quotient (E) and biochemical activities: dehydrogenase (F), β-glucosidase (G) and phosphatase (H). Letters on top of bars indicate significant differences (*P* < 0.05) for mean and standard error (*n* = 3). Cmic, microbial biomass carbon; PNP, p-nitrophenol, INTF, iodonitrotetrazolium formazan.

Direct observation of the roots showed that all *M. truncatula* plants were colonized by AMF. The mycorrhiza frequency (F%), which indicates the degree of root colonization by AMF, was significantly higher in samples from Sites 1 and 3 and lower in those from Sites 2 and 4 (Fig. 2). The pattern was even clearer when considering AMF colonization intensity (M%), defined as the percentage of roots colonized by AMF (Fig. 2).

The total number of AMF spores isolated from the rhizosphere of the studied plants (like in bulk soils, yet in smaller numbers and with no significant differences between Sites 1 and 3), varied significantly (*P* < 0.05) among the four sites, the highest value being recorded in Site 1 and the lowest in Site 4 (Fig. 2).

### Microbial and biochemical soil properties

Variations in microbial biomass (Cmic) followed the AMF colonization (F% and M%) results referred above (Fig. 2); similar results were observed in the metabolic quotient of bulk soil microbial communities, although without significant differences between Sites 1 and 2. However, distinct results were obtained for the metabolic quotient of the microbial communities from the rhizosphere soils, lower for Site 1 and higher for Site 4 (Fig. 2), whereas values for Sites 2 and 3 were not different from values observed for Sites 1 or 4.

The enzymatic activities (dehydrogenase, β-glucosidase and phosphatase activities) quantified in rhizosphere and bulk soils followed those observed for AMF colonization and microbial biomass (Fig. 2).

### AMF taxa and other organisms detected by 454-pyrosequencing

The BLAST search results against the NCBI database evidenced that the Glomeromycota only represented less than 20% of the total OTUs, in each one of the 12 samples (Table S2). In the root samples they represented 8–20% of the total OTUs. However, their detection in soil samples was restricted to rhizosphere soil from Site 1 (4%) and bulk soil from Sites 1 (5%), 2 (2%) and 3 (1%) (Table S2).

OTUs from other fungal groups were also detected: Basidiomycota (1–7%) in all samples, except roots from Sites 1 and 4; Chytridiomycota (2 and 1%), in the soil samples from Site 2; early diverging fungal lineages in all rhizosphere soil samples (0.9–1.4%) and root samples from Site 1 (0.5%) (Table S2).

A large fraction of the OTUs from the root samples of the four sites was included in the group “Other organisms” (33–44% of the OTUs found in each site), which is a broad range group including representatives of Eukaryota (Metazoa, Viridiplant, Ciliophora, Alveolata) and Prokaryota (Bacteria) (Table S2). Ascomycota represented 26–42% and both groups (Ascomycota and ”Other organisms”) represented 64–78% of the total OTUs found per site (Table S2). Concerning the rhizosphere and bulk soil samples, the groups Ascomycota and ”other organisms” represented 70–80% of the total OTUs in samples from Sites 1, 2 and 3, and 51–53% in samples from Site 4, where “Unidentified Eukaryota” and “Unidentified fungi” represented respectively 34–38% and 4–16% of the total OTUs per sample (Table S2).

Considering all the OTUs detected, the Chao I values obtained for the 12 samples subjected to 454-pyrosequencing varied between 74 and 333, the lowest value in the rhizosphere soil from Site 4 and the highest values in the rhizosphere soils from Sites 1 and 2 (333 and 332, respectively) (Table 3). Within each compartment, the lowest values were always found in Site 4 samples, which also presented the highest coverage values (Table 3) that reached more than 77% in all samples, except in the rhizosphere soil from Site 3 (65%) (Table 3).

### Glomeromycota identification based on the Maarj *AM* database

The distribution and sites of occurrence of the Glomeromycota OTUs with correspondences in the Maarj*AM* database is shown in Table S3. Based on matches with identity values ≥97%, 113 OTUs (82%), from a total of 138, found correspondences within 32 VTXs, two of them identified as species: *Scutellospora dipurpurescens* VTX49 (including two OTUs) and *Glomus coronatum* VTX265 (including 3 OTUs). The 32 identified VTXs comprised 5,743 matches in the Maarj*AM* database, differently distributed: from only 1 match (*Diversispora* sp. VTX355) up to 807 (*Glomus* sp. VTX113) (Table S3).

The OTUs that remained to be identified were grouped into 16 putative new taxa (pNTX) (Table S4). Only 10 OTUs (40%), included in seven pNTXs, matched with GenBank sequences with identity values higher than 97% (Table S5) and four pNTXs (integrating 10 OTUs) could not be assigned to a Glomeromycota family and remained identified as Glomeromycota sp. (Table S5).

The pNTXs were only detected in Sites 1, 2 and 3: nine in Site 1, four in Site 2 and four in Site 3; most were Site exclusive (Table S5).

**Table 3:** Chao1 index and Coverage values from the data generated by 454 pyrosequencing. Chao1 index and coverage values relative to the sequences and OTUs detected in roots and rhizosphere soils of *M. truncatula* and in bulk soils from the studied sites.

	Roots		Rhizosphere Soil		Bulk Soil	
	Chao1	Coverage	Chao1	Coverage	Chao1	Coverage
Site 1	209	80.86	333	78.91	257	86.03
Site 2	201	81.00	332	81.50	215	84.78
Site 3	249	84.48	107	64.85	171	77.32
Site 4	152	91.02	74	85.30	79	89.54

### AMF community composition and diversity

The 138 AMF OTUs were distributed by four families: 100 (72%) belong to the Glomeraceae, 15 (11%) to the Claroideoglomeraceae, 11 (8%) to the Diversisporaceae and 2 (2%) to the Gigasporaceae. Ten OTUs (7%) could not be assigned to an AMF family (Table 4).

**Table 4:** AMF families detected in all analyzed samples. Relative abundance of OTUs and corresponding taxa (VTXs and pNTXs) from each AMF family detected in roots and rhizosphere (Rhiz.) soils of *M. truncatula* and in bulk soils from the studied sites.

		Non-family assigned (Glomeromycota sp.)			Gigasporaceae			Diversisporaceae			Claroideoglomeraceae			Glomeraceae			TOTAL		
		VTX	pNTX	OTU	VTX	pNTX	OTU	VTX	pNTX	OTU	VTX	pNTX	OTU	VTX	pNTX	OTU	VTX	pNTX	OTU
Site 1	Roots		1	1				1		1	1		4	9	1	14	11	2	20
	Rhiz. Soil		1	1							1		1	3	5	11	4	6	13
	Bulk Soil		2	4										3	2	8	3	4	12
	**All samples**		**4**	**6**				**1**		**1**	**2**		**5**	**15**	**8**	**33**	**18**	**12**	**45**
																	**60%**	**40%**	
Site 2	Roots		2	2							1		3	11	2	22	12	4	27
	Rhiz. Soil																0	0	0
	Bulk Soil										1		4				1	0	4
	**All samples**		**2**	**2**							**2**		**7**	**11**	**2**	**22**	**13**	**4**	**31**
				** **			** **			** **			** **			** **	**76%**	**24%**	
Site 3	Roots		1	2	1		2	2		8	1		1	15	3	35	19	4	48
	Rhiz. Soil																0	0	0
	Bulk Soil													1	1	2	1	1	2
	**All samples**		**1**	**2**			**2**	**2**		**8**	**1**		**1**	**16**	**4**	**37**	**20**	**5**	**52**
				** **			** **			** **			** **			** **	**80%**	**20%**	
Site 4	Roots							2		2	2		2	7		8	11	0	12
	Rhiz. Soil																0	0	0
	Bulk Soil																0	0	0
	**All samples**							**2**		**2**	**2**		**2**	**7**		**8**	**11**	**0**	**12**
		** **	** **	** **	** **	** **	** **	** **	** **	** **	** **	** **	** **	** **	** **	** **	**100%**	**0%**	
**Global Values** (all Sites, all samples)				**10**			**2**			**11**			**15**			**100**	**62**	**21**	**138**
				**7%**			**2%**	** **	** **	**8%**	** **	** **	**11%**	** **	** **	**72%**	**75%**	**25%**	** **

**Table 5:** AMF taxa detected in *M. truncatula* root samples. Distribution of the 38 AMF taxa (29 VTX and 9 pNTX) detected in the root samples of *M. truncatula* from the four studied sites with indication of being Site exclusive (SE) or Non-Site exclusive (NE) and the corresponding numbers of OTUs.

		Roots				
		Site 1	Site 2	Site 3	Site 4	
Gigasporaceae	*S. dipurpurescens* VTX00049					
**Glomeraceae**	*Glomus* sp. VTX00256					SE
	*Glomus* sp. VTX00387					
	*Glomus* sp. pNTX P					
	*Glomus* sp. VTX00156					
	*Glomus* sp. VTX00166					
	*Glomus* sp. VTX00311					
	*Glomus* sp. pNTX H					
	*Glomus* sp. pNTX I					
	*Glomus* sp. VTX00065					
	*Glomus* sp. VTX00177					
	*Glomus* sp. VTX00199					
	*Glomus coronatum* VTX00265					
	*Glomus* sp. VTX00295					
	*Glomus* sp. VTX00307					
	*Glomus* sp. VTX00331					
	Glomus sp. pNTX E					
	*Glomus* sp. pNTX N					
	*Glomus* sp. pNTX O					
	*Glomus* sp. VTX00151					NE
	*Glomus* sp. VTX00342					
	*Glomus* sp. VTX00067					
	*Glomus* sp. VTX00092					
	*Glomus* sp. VTX000113					
	*Glomus* sp. VTX00280					
	*Glomus* sp. VTX00105					
	*Glomus* sp. VTX00108					
	*Glomus* sp. VTX00114					
	*Glomus* sp .VTX00115					
Diversisporaceae	*Diversispora* sp. VTX00054					SE
	*Diversispora* sp. VTX00380					
	*Diversispora* sp. VTX00377					
	*Diversispora* sp. VTX00355					NE
Claroideoglo meraceae	*Claroideoglomus* sp. VTX00193					
	*Claroideoglomus* sp. VTX00357					SE
Non-family assigned	Glomeromycota sp. pNTX K					
	Glomeromycota sp. pNTX A					
	Glomeromycota sp. pNTX C					NE
Number	VTX	11	12	19	11	
	pNTX	2	4	4	0	
	OTUs	20	27	48	12	

The four families were found in the 107 AMF OTUs detected in all root samples (Tables 4 and 5). However, differences in the AMF communities were observed between sites, the four families being present only in root samples from Site 3, where the unique VTX belonging to the Gigasporaceae was detected (Table 5). The AMF OTUs from Site 1 belong to three families (Glomeraceae, Claroideoglomeraceae and Diversisporaceae), while Site 2 and Site 4 presented less AMF diversity, with only two families detected (Glomeraceae and Claroideoglomeraceae); the “Non-family assigned” OTUs were not detected in Site 4 root samples (Tables 4 and 5).

**Table 6:** AMF taxa detected in soil samples: bulk soil and *M. truncatula* rhizosphere soil. Distribution of the 15 AMF taxa (six VTX and nine pNTX) identified in the rhizosphere soils of *M. truncatula* and in the bulk soils from the studied sites, with indication of being Site exclusive (SE) or Non-site Exclusive (NE) and the corresponding numbers of OTUs.

	Rhizosphere soil					Bulk soil				
	Site 1	Site 2	Site 3	Site 4		Site 1	Site 2	Site 3	Site 4	
Claroideoglomeraceae					*Claroideoglomus* sp. VTX00193					NE
Glomeraceae					*Glomus* sp. VTX00151					SE
					*Glomus* sp. VTX00419					
					*Glomus* sp. pNTX D					
					*Glomus* sp. VTX309					
					*Glomus* sp. pNTX B					
					*Glomus* sp. pNTX E					
					*Glomus* sp. pNTX G					
					*Glomus* sp. pNTX L					
					*Glomus* sp. VTX98					
					*Glomus* sp. pNTX J					
					*Glomus* sp. VTX342					
					*Glomus* sp. pNTX M					
Non-family assigned					Glomeromycota sp. pNTX C					
					Glomeromycota sp. pNTX F					
VTX	4	0	0	0		3	1	1	0	VTX
pNTX	6	0	0	0	Number	4	0	1	0	pNTX
OTUs	13	0	0	0		12	4	1	0	OTUs

In respect to the 31 OTUs from soil samples (Tables 4 and 6), 13 were detected in rhizosphere soil from Site 1, being distributed by two families: Glomeraceae (3 VTX and 6 pNTX) and Claroideoglomeraceae (1 VTX). The other OTUs were detected in bulk soil samples: 12 OTUS (3 VTX and 4 pNTX) in Site 1 and two (1 VTX and 1 pNTX) in Site 3 belong to the Glomeraceae, while four OTUs detected in Site 2 were included in one VTX belonging to the Claroideoglomeraceae; “Non-family assigned” OTUs were only detected in Site 1, both in rhizosphere and in bulk soil samples (Tables 4 and 6).

Considering the AMF genera detected in this study, *Glomus* was predominant in the four studied sites, *Glomus* and *Claroideoglomus* being common genera to all sites (Tables 5–7). *Scutellospora* (VTX49) was only detected in the high-altitude site (Site 3; Table 5).

**Table 7:** Non-Site exclusive taxa and global distribution of the identified taxa. Distribution of the Non-Site exclusive (NE) taxa detected in this study. The SE taxa were identified in Figures 3 and 4. All NE taxa were found in the root samples from all the sites of detection, except Glomus sp. pNTX E (in Site 1) and *Glomus* sp. VTX00342 (in Site 3).

Non-Site exclusive taxa	Site 1	Site 2	Site 3	Site 4
*Claroideoglomus* sp. VTX00193				
*Glomus* sp. VTX00105				
*Glomus* sp. VTX00108				
*Glomus* sp. VTX00114				
*Glomus* sp .VTX00115				
*Glomus* sp. VTX000113				
*Glomus* sp. VTX00280				
*Glomus* sp. VTX00151				
Glomeromycota sp. pNTX C				
*Glomus* sp. pNTX E				
*Diversispora* sp. VTX00355				
*Glomus* sp. VTX00342				
*Glomus* sp. VTX00067				
*Glomus* sp. VTX00092				
Number of Non-Site exclusive taxa	11	10	11	9
Number of Site exclusive taxa	15	6	14	2
TOTAL	26	16	25	11

Considering the Site-distribution of the VTXs and pNTXs detected in this study, from the 14 Non-Site exclusive taxa (12 VTX and 2 pNTX), five (36%) were common to all sites, where they varied between nine in Site 4 and 11 in Sites 1 and 3 (Table 7; all these taxa were found in root samples while only five were found in soil samples (Tables 5 and 6). Overall, pNTXs correspond to 25% of the total taxa (40% in Site 1 and 0% in Site 4) (Table 4).

A week percentage of exclusive taxa was observed in Sites 2 and 4 (only 38 and 18% of the total AMF taxa, respectively) while they represented more than 50%, in Sites 1 and 3 (Table 7).

### Global evaluation of the AMF diversity within the four studied sites

Sites 1 and 3 presented the highest diversity of AMF taxa (VTX and pNTX), considering all the samples from the three compartments (roots, rhizospheres and bulk soil), with high evenness (Tables 5 and 6). Shannon and Simpson indexes were also higher in these two sites (Table 8). AMF genetic evenness (Pielou’s evenness index) indicated that all taxa were equitably present in all sites, notably in Site 4.

**Table 8:** Shannon, Simpson and Pielou’s evenness indexes for the AMF taxa (VTX and pNTX) detected in root, rhizosphere and bulk soil samples from the four studied sites.

	Shannon index (H)	Simpson index (D)	Pielou’s evenness index (J)
Site 1	2.91	15.33	0.92
Site 2	2.54	9.90	0.91
Site 3	2.90	12.25	0.92
Site 4	2.36	10.28	0.98

As indicated by the Jaccard’s index values and considering all the samples of the three compartments from each site, the highest similarity in the AMF taxa (VTX and pNTX) was detected in Sites 1 and 2 (0.30), followed by Sites 2 and 4 (0.28); the least similar were Sites 1 and 3 (0.18), followed by Sites 1 and 4 (0.21) due to Site exclusive AMF taxa found in those sites (Table 9).

**Table 9:** Pairwise comparison of the sites based on the AMF taxa (VTX and pNTX) detected in root, rhizosphere and bulk soil samples, according to the Jaccard similarity index.

Site	1	2	3	4
1	–	0.30	0.18	0.21
2	–	–	0.22	0.28
3	–	–	–	0.25
4	–	–	–	–

Differences in AMF diversity and richness between the four sites, were significantly affected by the major environmental parameters evaluated (*p* values ≤ 0.023), as indicated in Table 10, except for pH (*p*-values of 0.144 and 0.451, respectively) and altitude level (*p*-values of 0.462 and 0.214, respectively). Across the sampled sites, strong correlations were found between AMF communities’ composition (diversity and richness) and two soil parameters: total phosphorus (*z* =  − 2.594; *p* < 0.001 and *z* =  − 4.704; *p* < 0.001 respectively) and texture (*z* =  − 3.015; *p* < 0.001 and *z* =  − 4.807; *p* < 0.001 respectively).

**Table 10:** Logistic Regression analysis to assess the relevance of environmental parameters in predicting the AMF composition and diversity.

Environmental parameters	AMF taxa		AMF OTUs	
	*z*-score	*p*-value	*z*-score	*p*-value
pH	−1.460	0.144^ns^	0.753	0.451^ns^
Electrical conductivity (E.c)	3.015	0.002^**^	4.807	0.000^***^
Organic matter (Org.m)	3.053	0.002^**^	2.259	0.023^*^
Total nitrogen (T.N)	3.129	0.001^**^	3.180	0.001^**^
Total phosphorus (T.P)	−2.594	0.009^**^	−4.704	0.000^***^
Grazing intensity	2.388	0.016^*^	3.889	0.000^***^
Texture	−3.015	0.002^**^	−4.807	0.000^***^
Altitude	0.736	0.462^ns^	1.242	0.214^ns^

**Notes.**

ns No significant effect (*p* > 0.05) * Significant effect at *p* < 0.05 ** Significant effect at *p* < 0.01 *** Significant effect at *p* < 0.001

**Figure 3:**
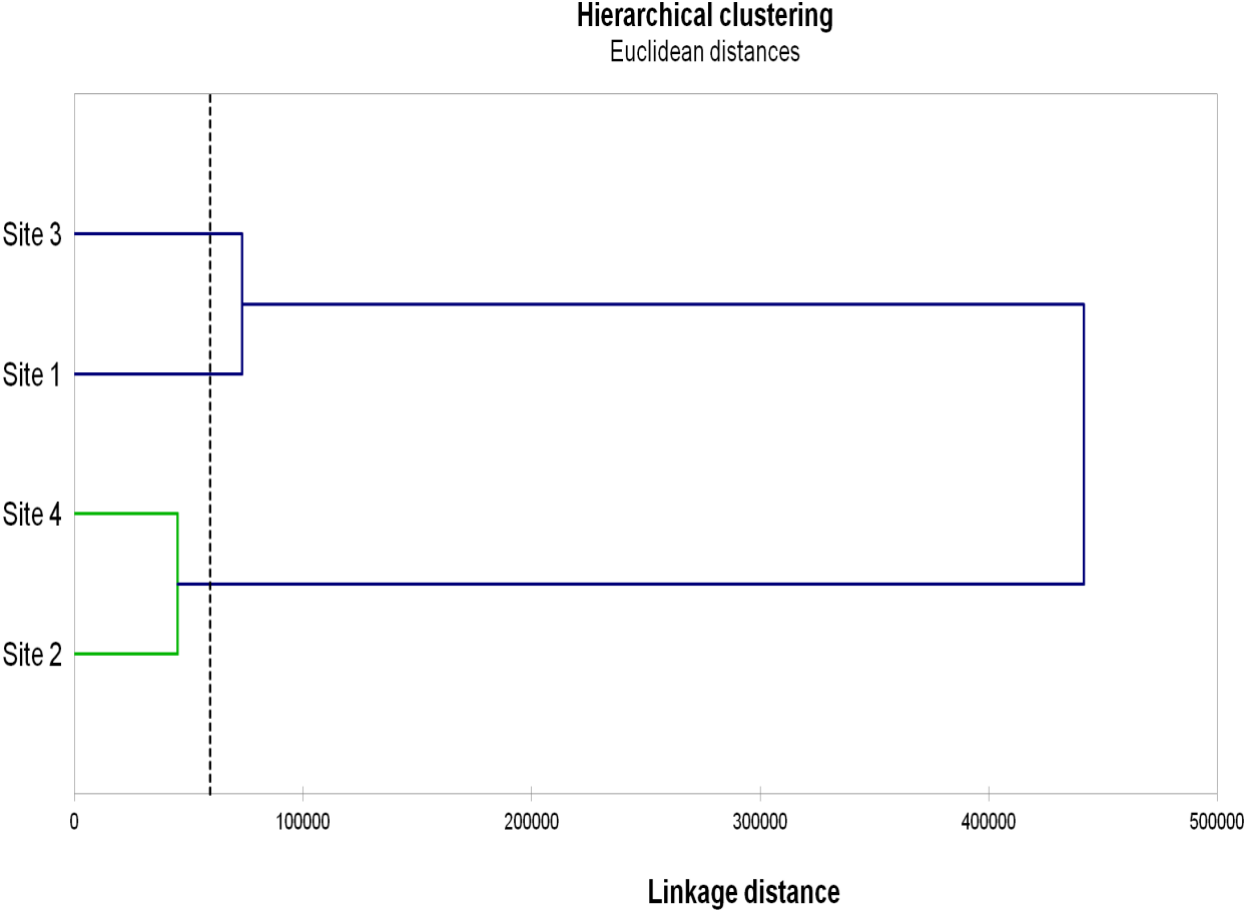
Hierarchical clustering of the studied sites. Hierarchical clustering of the studied sites based on the AMF diversity (total numbers of OTU, VTX and pNTX), AMF colonization (mycorrhizal frequency, mycorrhizal intensity, spore density), microbial parameters (microbial biomass, metabolic quotient) and biochemical activities (dehydrogenase, β-glucosidase, phosphatase) evaluated in root, rhizosphere and bulk soil samples from the studied sites.

The results of Hierarchical clustering (Fig. 3) grouped Sites 2 and 4 and, although more distantly, Sites 1 and 3.

## Discussion

In this study the data concerning AMF molecular identifications are not suitable for statistical comparison of AMF diversity between the studied sites because we analyzed one composite sample per compartment and per site. Thus, the diversity detected in each composite sample corresponds to three independent amplifications performed on three separate samples, whose products contributed in equimolar concentrations to a final pyrosequencing reaction. According to the Chao1 values obtained, every sequence present in the pyrosequencing process, was eventually amplified. Thus, the obtained AMF community data can be used to describe variations in AMF diversity and composition between study sites and in response to soil parameters.

### Primers selectivity and Glomeromycota discrimination

Most of the published AMF diversity data has been obtained targeting the small subunit (SSU) of the *rRNA* gene with the primer set used in this study, among others (Lee, Lee & Young, 2008). This was the case of the study of Öpik et al. (2009) which, using 454 pyrosequencing, was the first to detect and identify AMF communities from environmental samples.

A first BLAST search against the NCBI database showed, for the 2,320 OTUs generated by pyrosequencing, that AMF were not the most abundant organisms though better represented in root than in soil samples. Most OTUs belonged to Ascomycota or “Other Organisms” and, in lower percentages, to other fungal phyla. The high percentage of “unknown” taxa (comprising “Unidentified Eukaryota” and “Unidentified fungi”) was surprising and possibly represent not yet assigned taxa, including Glomeromycota, specific of this type of environment. In fact, it has been suggested that AMF diversity is far from adequately described (Öpik et al., 2009), and it is possible that some unexplored ecosystems harbor many unknown AMF species (Liu et al., 2011). Also supporting this suggestion was the high percentage of pNTX found in this study: 33% of the total identified taxa (VTX and pNTX).

Our results also showed that the primer set selected as AMF specific had a broader spectrum of detection, allowing the amplification of both Eukaryota and Prokaryota sequences. Co-amplification of plant and other organisms’ DNA with this primer set was already referred by Alguacil et al. (2011) and Van Geel et al. (2014), among other authors. Van Geel et al. (2014) demonstrated the higher specificity of another primer set (NS31/AML2) that rendered, in a study also conducted in a semi-arid region, about 43% of Glomeromycota reads (Varela-Cervero et al., 2015).

### Patterns of AM fungal community composition

BLAST searches against the recognized and curated Maarj*AM* database (Öpik et al., 2010) allowed the identification of 113 AMF OTUs as 32 VTX while 25 OTUs were grouped in 16 putative new taxa (pNTX). In a study conducted with the same primer set (Varela-Cervero et al., 2015) new AMF taxa were also reported colonizing five plant species of a Mediterranean semi-arid region, which highlights the need for more work focusing AMF diversity in this type of ecosystems.

From all the detected genera, only *Glomus* and *Claroideoglomus* were found in all studied sites. *Glomus* has been described as the dominant genus in AMF assemblages, which may be explained by its ability to produce large numbers of spores and hypha fragments, thus enabling it to be better adapted to drastic conditions (Öpik et al., 2006; Zhao et al., 2017); it is also known as the most ubiquitous and stress/perturbation tolerant (Öpik et al., 2009), having been referred as the major AMF group present in Mediterranean degraded semi-arid areas (Alguacil et al., 2011; Gomes et al., 2015) and the most resistant and adapted to semi-arid regions (Zhao et al., 2017; Mosbah, Philippe & Mohamed, 2018). There are also references that alkaline pH significantly influences the dominant distribution of *Glomus* (Xiang et al., 2014; Mosbah, Philippe & Mohamed, 2018). Since both conditions co-exist in the studied sites, our results are in accordance with these reports.

*Claroideoglomus* has been referred to increase root length and to improve nutrients uptake and the compatibility between AMF and host plants (Liu, Srivastava & Wu, 2017). These roles are, eventually, of major importance in semi-arid conditions.

On the other end, Gigasporaceae (*S. dipurpurescens*) was exclusively detected in Site 3 and Diversisporaceae (*Diversispora* sp.) was detected in all sites except Site 2; pNTXs, including Non-family assigned Glomeromycota, were detected in all but Site 4. Accordingly, Sites 4 and 2 presented the lowest AMF diversity and Site-exclusive AMF taxa; they were also characterized by lower values of AMF colonization, and microbial and biochemical activities, clustering together in a hierarchal analysis based on those and other site characteristics. The same analysis clustered Sites 1 and 3. It is interesting that Site clustering grouped soils with more similar pH, electrical conductivity, organic matter and consequently higher phosphorus and nitrogen concentration, factors already know as key players in determining AMF diversity.

### Patterns of AM fungal diversity

The number of identified taxa (VTXs and pNTXs) varied in a consistent manner among the three compartments from each site, from only a few in soil samples (0 in Site 4 soil as well as in most rhizosphere soils) to a minimum of 11 in root samples. Similar results have been reported, showing higher AMF diversity associated with plant roots than with soil samples (e.g., (Öpik et al., 2009). Since a goal of this study was the comparison of the AMF found in the four sites, the taxa from the three compartments of each site were considered together for calculations of AMF diversity (number of taxa) and richness (number of OTUs).

Considering the numbers of distinct taxa (including VTX and pNTX), Sites 1 and 3 presented higher AMF diversity than Sites 2 and 4, which is in accordance with the corresponding Shannon index values. Worth noticing that the Shannon index indicated that the four sites were very diverse (Mosbah, Philippe & Mohamed, 2018), even in comparison with less stressed environments (Gai et al., 2009). Recent studies, in line with our results, showed perturbation and not stress as a main factor affecting AMF diversity in natural ecosystems (Öpik et al., 2008; Bonfim, Vasconcellos & Gumiere, 2016). The equitable distribution of the AMF taxa among sites, based on Pielou’s evenness index, did not reflect the management intensity distinctive of Site 4, which suggests that plant roots (the hotspots of AMF diversity) were not significantly affected by the associated perturbations (e.g., grazing).

Comparing the sites based on shared AMF taxa (Jaccard’s index), the most similar ones only shared 30% of the AMF species, suggesting that the distribution of AM fungal communities depend on site properties (Lekberg et al., 2011; Gosling et al., 2013). It is worth stressing such large variations in the AMF colonizing the same plant species in a relatively small and geographically uniform area with the same aridity index. This clearly shows that AMF communities are influenced not only by the host plant, but also by local environmental factors, besides aridity level (Coutinho et al., 2015; Velázquez et al., 2016).

### Links between environmental condition and AM fungal diversity

Soil properties have been considered important factors determining AMF community composition (Oehl et al., 2010). This was confirmed in our study, since significative correlations were observed between the number of AMF taxa and AMF OTUs and six of the seven soil parameters evaluated in this study. High total nitrogen concentration, soil organic matter and electrical conductivity positively influenced AMF diversity, which was especially evident for Site 1. Identical correlations were found by Helgason et al. (1998) and Oehl et al. (2010). These results are in accordance with the AMF influence on soil biochemical reactions, including mineralization of organic matter and nitrification (Hamel, 2004). The grazing activity (more intensive in Site 4) may modify soil biological properties (Fterich, Mahdhi & Mars, 2012) and decrease AMF diversity, as observed in this study. However other characteristics may have contributed to the lower AMF diversity of Site 4, such as soil texture, pH (Zhao et al., 2017) or higher phosphorus (P) availability (Liu et al., 2016). P availability modifies the partitioning of resources in AMF propagules thus reducing the genetic diversity of AMF in soil (Sheng et al., 2012).

Altitude may also affect AMF diversity (Gai et al., 2009) but this correlation was not found in this study. The same (no significant correlation) was recorded for pH which is known to significantly influences AMF diversity (Mosbah, Philippe & Mohamed, 2018); this may be due to the minor differences observed between sites, all of them with alkaline pH.

Significant differences between sites could be observed in the AMF colonization of plant roots and in spore density in the rhizosphere soils, as well as in the microbial biomass and enzymatic activities evaluated in the rhizosphere soils, confirming the important roles that AMF play in soil and microbial processes (Rillig, 2004). Consistently, the highest microbial activity was associated with Site 1, followed by Site 3. AMF are known to affect the microbial community composition in the rhizosphere of the host plant (Johansson, Paul & Finlay, 2004) and this may be explained by the multitude of organic compounds released by mycorrhizal roots, making the rhizosphere a hot spot of microbial activities (Asmelash, Bekele & Birhane, 2016).

Since the microbial respiration per unit microbial biomass depends on C:N ratio of the substrate (Spohn, 2015), the huge increase of the metabolic quotient from the rhizosphere to the soil reflects the importance of the vegetation in determining soil characteristics (through their root microbiome) and in particular in promoting soil carbon sequestration. This hypothesis was supported by the higher similarity of the metabolic quotient of the rhizosphere in relation to the soil compartments of the four sites. The observed differences in the microbial metabolic quotients among compartments are possibly due to their distinct microbial communities (Fterich, Mahdhi & Mars, 2012), a result that highlights the correlation between soil ecology and soil functionality (Xu et al., 2014).

### Conclusion

Considering that AMF colonization is an important factor for plant fitness and that our results were obtained when AMF were well established in the sampled plants, they may indicate consistent and well-functioning AM symbiosis in *M. truncatula* roots from each studied site. The highest AMF diversity was observed in Sites 1 and 3 that corresponded to a higher AMF genetic variation and may provide higher functional diversity, since it is accepted that phylogenetically distant AMF may have more distinct effects in their host plants than more closely related ones. Interaction with more diverse AMF communities may increase plants growth and better adaptation to their fragile ecosystems (Powell et al., 2009; Ehinger et al., 2012) i.e., a semi-arid region.

Our results are consistent with the idea that host plants can select AMF species in response to local properties. To our best knowledge, this work reveals an unexpected diversity and heterogeneity of the AMF colonization of a plant species—*M. truncatula*—under semi-arid conditions and highlights AMF importance as a tailored mechanism of plant adaptation to local environmental conditions.

These findings revealed that under identical severe aridity conditions within the same region, and evaluated for the same host plant, AMF diversity can vary substantially in relation with environmental factors.

##  Supplemental Information

10.7717/peerj.6401/supp-1 Table S1 Glomeromycota taxa detected in roots and rhizosphere soils of *M. truncatula* and in bulk soils from the four studied sites The identifications were based on BLAST searches against the Maarj*AM* database (http://maarjam.botany.ut.ee). The OTUs without correspondence to a virtual taxon (VTX) in the Maarj*AM* database (or presenting correspondences with identity values <97%) were identified as putative new taxa (pNTX), each one comprising OTUs sharing pairwise distance values <3%. **SP1**: Plant from Site 1; **SP2**: Plant from Site 2; **SP3**: Plant from Site 3; **SP4**: Plant from Site 4. **SS1**: Rhizosphere soil from Site 1; **SS2**: Rhizosphere soil from Site 2; **SS3**: Rhizosphere soil from Site 3; **SS4**: Rhizosphere soil from Site 4. **SCS1**: Bulk soil from Site 1; **SCS2**: Bulk soil from Site 2; **SCS3**: Bulk soil from Site 3; **SCS4**: Bulk soil from Site 4.

10.7717/peerj.6401/supp-2 Table S2 Identified organisms in the OTUs generated by 454 pyrosequencing Number (#) and percentage (%) of OTUs from each specified group of organisms, detected in roots and rhizosphere (Rhiz) soils of *M. truncatula* and in bulk soils, from the studied sites. *- Includes representatives of Eukaryota (Metazoa, Viridiplant, Ciliophora, Alveolata) and Prokaryota (Bacteria).

10.7717/peerj.6401/supp-3 Table S3 Virtual taxa (VTX) identified in the samples subjected to 454 pyrosequencing Virtual taxa (VTX) from the Maarj*AM* database with correspondence to the AMF OTUs identified in the DNA samples from *M. truncatula* roots and rhizosphere soils and from the bulk soils subjected to 454 pyrosequencing directed to the SSU rDNA region.

10.7717/peerj.6401/supp-4 Table S4 Pairwise distances matrix generated to establish putative new taxa Pairwise distances matrix generated to constitute putative new taxa (pNTX) that group together OTU sequences sharing distances ≤3%.

10.7717/peerj.6401/supp-5 Table S5 Putative new taxa (pNTX) identified in the samples subjected to 454 pyrosequencing AMF OTUs without significant identity (≥97%) with taxa from the *Maar* j*AM* database and subjected to pairwise distance evaluation for the establishment of putative new taxa (pNTX). Each pNTX is either a single OTU or OTUs sharing distances ≤0.03.

10.7717/peerj.6401/supp-6 File S1 Raw data: Full Taxon Report for the SSU region. **SP1**: Plant from Site 1; **SP2**: Plant from Site 2; **SP3**: Plant from Site 3; **SP4**: Plant from Site 4. **SS1**: Rhizosphere soil from Site 1; **SS2**: Rhizosphere soil from Site 2; **SS3**: Rhizosphere soil from Site 3; **SS4**: Rhizosphere soil from Site 4. **SCS1**: Bulk soil from Site 1; **SCS2**: Bulk soil from Site 2; **SCS3**: Bulk soil from Site 3; **SCS4**: Bulk soil from Site 4.

## Additional Information and Declarations

### Field Study Permissions

The following information was supplied relating to field study approvals (i.e., approving body and any reference numbers): Field experiments were approved by the Forestry Service of the Minister of Agriculture of Tunisia Republic (Permit 1043).

### DNA Deposition

The following information was supplied regarding the deposition of DNA sequences: The SSU sequences from AMF OTUs are available under accession numbers MG321415 –MG321557.

### Data Availability

The following information was supplied regarding data availability: NCBI, SRA database with the following accession: SRP153928.
