# Supplementary material for: Arbuscular mycorrhizal fungi in soil, roots and rhizosphere of Medicago truncatula: diversity and heterogeneity under semi-arid conditions
Source: PeerJ. 2019 Mar 1;7:e6401. doi: 10.7717/peerj.6401 (PMC6398376; doi:10.7717/peerj.6401)
Supplement: Table S1 — The identifications were based on BLAST searches against the MaarjAM database (http://maarjam.botany.ut.ee). The OTUs without correspondence to a virtual taxon (VTX) in the MaarjAM database (or presenting correspondences with identity values <97%) were identified as putative new taxa (pNTX), each one comprising OTUs sharing pairwise distance values <3%. SP1: Plant from Site 1; SP2: Plant from Site 2; SP3: Plant from Site 3; SP4: Plant from Site 4. SS1: Rhizosphere soil from Site 1; SS2: Rhizosphere soil from Site 2; SS3: Rhizosphere soil from Site 3; SS4: Rhizosphere soil from Site 4. SCS1: Bulk soil from Site 1; SCS2: Bulk soil from Site 2; SCS3: Bulk soil from Site 3; SCS4: Bulk soil from Site 4. [file peerj-07-6401-s001.docx]

| **Family** | OTU | GenBank accession number | Maarj*AM* virtual taxon (VTX) / putative new taxon (pNTX) |
| --- | --- | --- | --- |
| **Gigasporaceae** | 4YP04H7RNY_SP3 | MG321509 | *Scutellospora* *dipurpurescens* VTX00049 |
|  | 4YP04JR2H7_SP3 | MG321510 | *Scutellospora dipurpurescens* VTX00049 |
| **Diversisporaceae** | 4YP04H3IP4_SP1 | MG321415 | *Diversispora* sp*.*VTX00355 |
|  | 4YP04IA4J4_SP3 | MG321463 | *Diversispora* sp. VTX00380 |
|  | 4YP04I6XFH_SP3 | MG321464 | *Diversispora* sp. VTX00054 |
|  | 4YP04JUWOQ_SP3 | MG321465 | *Diversispora* sp. VTX00054 |
|  | 4YP04I5AYZ_SP3 | MG321478 | *Diversispora* sp. VTX00054 |
|  | 4YP04I5JV2_SP3 | MG321479 | *Diversispora* sp. VTX00380 |
|  | 4YP04IOW4L_SP3 | MG321480 | *Diversispora* sp. VTX00054 |
|  | 4YP04IX19T_SP3 | MG321481 | *Diversispora* sp. VTX00054 |
|  | 4YP04IWVZT_SP3 | MG321483 | *Diversispora* sp. VTX00380 |
|  | 4YP04JWWD9_SP4 | MG321511 | *Diversispora* sp. VTX00355 |
|  | 4YP04IMZBC_SP4 | MG321513 | *Diversispora* sp. VTX00377 |
| **Claroideoglomeraceae** | 4YP04JEEPO_SP1 | MG321416 | *Claroideoglomus* sp. VTX00193 |
|  | 4YP04JI4UB_SP1 | MG321427 | *Claroideoglomus* sp. VTX00193 |
|  | 4YP04IUURF_SP1 | MG321428 | *Claroideoglomus* sp. VTX00193 |
|  | 4YP04IYQRH_SP1 | MG321433 | *Claroideoglomus* sp. VTX00193 |
|  | 4YP04I72AD_SP2 | MG321435 | *Claroideoglomus* sp. VTX00193 |
|  | 4YP04INILH_SP2 | MG321445 | *Claroideoglomus* sp. VTX00193 |
|  | 4YP04JCI3Q_SP2 | MG321458 | *Claroideoglomus* sp. VTX00193 |
|  | 4YP04JRWZS_SP3 | MG321503 | *Claroideoglomus* sp. VTX00193 |
|  | 4YP04IC9XZ_SP4 | MG321516 | *Claroideoglomus* sp. VTX00357 |
|  | 4YP04I676Z_SP4 | MG321518 | *Claroideoglomus* sp. VTX00193 |
|  | 4YP04HZP6O_SS1 | MG321536 | *Claroideoglomus* sp. VTX00193 |
|  | 4YP04IU7FM_SCS2 | MG321551 | *Claroideoglomus* sp. VTX00193 |
|  | 4YP04JSD57_SCS2 | MG321552 | *Claroideoglomus* sp. VTX00193 |
|  | 4YP04JFOLF_SCS2 | MG321553 | *Claroideoglomus* sp. VTX00193 |
|  | 4YP04JOOD0_SCS2 | MG321554 | *Claroideoglomus* sp. VTX00193 |
| **Glomeraceae** | 4YP04JRRNN_SP1 | MG321417 | *Glomus* sp. VTX00108 |
|  | 4YP04H7NF6_SP1 | MG321418 | *Glomus* sp. VTX00115 |
|  | 4YP04JJ9IX_SP1 | MG321419 | *Glomus* sp. VTX00113 |
|  | 4YP04I6O9X_SP1 | MG321420 | *Glomus* sp. VTX00113 |
|  | 4YP04ICWLW_SP1 | MG321421 | *Glomus* sp. VTX00113 |
|  | 4YP04JPHQV_SP1 | MG321422 | *Glomus* sp. VTX00256 |
|  | 4YP04JXDM6_SP1 | MG321423 | *Glomus* sp. VTX00280 |
|  | 4YP04IFHAF_SP1 | MG321424 | *Glomus* sp. VTX00115 |
|  | 4YP04IHMFP_SP1 | MG321425 | *Glomus* sp. pNTX P |
|  | 4YP04JJ20Y_SP1 | MG321426 | *Glomus* sp. VTX00114 |
|  | 4YP04JOJ2U_SP1 | MG321429 | *Glomus* sp. VTX00387 |
|  | 4YP04IUH19_SP1 | MG321430 | *Glomus* sp. VTX00115 |
|  | 4YP04IR2P0_SP1 | MG321431 | *Glomus* sp. VTX00151 |
|  | 4YP04IQ20K_SP1 | MG321434 | *Glomus* sp. VTX00105 |
|  | 4YP04JRAU1_SP2 | MG321436 | *Glomus* sp*.* VTX00113 |
|  | 4YP04JCJR6_SP2 | MG321437 | *Glomus* sp*.* VTX00113 |
|  | 4YP04IGZBS_SP2 | MG321438 | *Glomus* sp*.* VTX00114 |
|  | 4YP04IJRDF_SP2 | MG321439 | *Glomus* sp*.* VTX00113 |
|  | 4YP04I54EP_SP2 | MG321440 | *Glomus* sp*.* VTX00115 |
|  | 4YP04H05UY_SP2 | MG321441 | *Glomus* sp. VTX00108 |
|  | 4YP04II2S8_SP2 | MG321442 | *Glomus* sp*.* VTX00115 |
|  | 4YP04JIKA1_SP2 | MG321443 | *Glomus* sp*.* VTX00114 |
|  | 4YP04IPD87_SP2 | MG321444 | *Glomus* sp. VTX00105 |
|  | 4YP04JJQKI_SP2 | MG321446 | *Glomus* sp.VTX00105 |
|  | 4YP04JCB34_SP2 | MG321447 | *Glomus* sp. pNTX I |
|  | 4YP04H7PR2_SP2 | MG321448 | *Glomus* sp*.* VTX00342 |
|  | 4YP04I5YAR_SP2 | MG321449 | *Glomus* sp*.* VTX00342 |
|  | 4YP04IRZFU_SP2 | MG321450 | *Glomus* sp*.* VTX00156 |
|  | 4YP04I0PYT_SP2 | MG321451 | *Glomus* sp. pNTX H |
|  | 4YP04HZG3E_SP2 | MG321452 | *Glomus* sp*.* VTX00151 |
|  | 4YP04JA80E_SP2 | MG321453 | *Glomus* sp*.* VTX00311 |
|  | 4YP04JA7WM_SP2 | MG321454 | *Glomus* sp*.* VTX00166 |
|  | 4YP04I92QC_SP2 | MG321455 | *Glomus* sp*.* VTX00114 |
|  | 4YP04H2GXM_SP2 | MG321456 | *Glomus* sp. VTX00108 |
|  | 4YP04I453R_SP2 | MG321460 | *Glomus* sp*.* VTX00280 |
|  | 4YP04JLR0M_SP2 | MG321461 | *Glomus* sp*.* VTX00115 |
|  | 4YP04JIX0M_SP3 | MG321462 | *Glomus* sp. VTX00177 |
|  | 4YP04JJ9K2_SP3 | MG321466 | *Glomus* sp. VTX00067 |
|  | 4YP04JLI5E_SP3 | MG321467 | *Glomus* sp*.* VTX00067 |
|  | 4YP04IU15P_SP3 | MG321468 | *Glomus coronatum* VTX00265 |
|  | 4YP04H5E7K_SP3 | MG321469 | *Glomus* sp. VTX00067 |
|  | 4YP04IIFRH_SP3 | MG321470 | *Glomus* sp. VTX00307 |
|  | 4YP04IW882_SP3 | MG321471 | *Glomus* sp. pNTX E |
|  | 4YP04I0HTO_SP3 | MG321473 | *Glomus* sp. pNTX O |
|  | 4YP04H9LJ6_SP3 | MG321474 | *Glomus* *coronatum* VTX00265 |
|  | 4YP04H4GDP_SP3 | MG321475 | *Glomus* sp. VTX00067 |
|  | 4YP04IKHE2_SP3 | MG321476 | *Glomus* sp. VTX00295 |
|  | 4YP04JQ0KZ_SP3 | MG321477 | *Glomus* sp. VTX00331 |
|  | 4YP04IG7YE_SP3 | MG321484 | *Glomus* sp. VTX00114 |
|  | 4YP04IJZVD_SP3 | MG321485 | *Glomus* sp. VTX00113 |
|  | 4YP04JTD44_SP3 | MG321487 | *Glomus* sp. VTX00105 |
|  | 4YP04I432E_SP3 | MG321488 | *Glomus* sp. VTX00108 |
|  | 4YP04JR9ZY_SP3 | MG321489 | *Glomus* sp. VTX00331 |
|  | 4YP04IOER2_SP3 | MG321490 | *Glomus* sp. VTX00113 |
|  | 4YP04I9AWX_SP3 | MG321491 | *Glomus* sp. VTX00065 |
|  | 4YP04ILY5Z_SP3 | MG321492 | *Glomus* sp. VTX00067 |
|  | 4YP04ITJN7_SP3 | MG321493 | *Glomus* sp. VTX00092 |
|  | 4YP04JQ0V0_SP3 | MG321494 | *Glomus* *coronatum* VTX00265 |
|  | 4YP04IN9BR_SP3 | MG321495 | *Glomus* sp. VTX00115 |
|  | 4YP04IVWAC_SP3 | MG321496 | *Glomus* sp. VTX00067 |
|  | 4YP04I6OPZ_SP3 | MG321497 | *Glomus* sp. VTX00115 |
|  | 4YP04I24NN_SP3 | MG321498 | *Glomus* sp. VTX00067 |
|  | 4YP04H8FTL_SP3 | MG321499 | *Glomus* sp. VTX00105 |
|  | 4YP04JVBOQ_SP3 | MG321500 | *Glomus* sp. VTX00067 |
|  | 4YP04IVQAN_SP3 | MG321501 | *Glomus* sp. VTX00280 |
|  | 4YP04IU4ZQ_SP3 | MG321502 | *Glomus* sp. pNTX N |
|  | 4YP04JU288_SP3 | MG321504 | *Glomus* sp. VTX00199 |
|  | 4YP04I5FQK_SP3 | MG321505 | *Glomus* sp. VTX00199 |
|  | 4YP04ILIUP_SP3 | MG321506 | *Glomus* sp*.* VTX00067 |
|  | 4YP04IH0CT_SP3 | MG321507 | *Glomus* sp. VTX00065 |
|  | 4YP04II1LM_SP3 | MG321508 | *Glomus* sp. VTX00108 |
|  | 4YP04JDUFC_SP4 | MG321512 | *Glomus* sp. VTX00067 |
|  | 4YP04IF6FZ_SP4 | MG321514 | *Glomus* sp. VTX00115 |
|  | 4YP04I9T57_SP4 | MG321515 | *Glomus* sp. VTX00105 |
|  | 4YP04I5G3I_SP4 | MG321517 | *Glomus* sp. VTX00108 |
|  | 4YP04ITJ5A_SP4 | MG321519 | *Glomus* sp. VTX00342 |
|  | 4YP04JLNL6_SP4 | MG321520 | *Glomus* sp. VTX00114 |
|  | 4YP04IE0YL_SP4 | MG321521 | *Glomus* sp. VTX00342 |
|  | 4YP04I6M3Y_SP4 | MG321522 | *Glomus* sp. VTX00092 |
|  | 4YP04H25BH_SS1 | MG321523 | *Glomus* sp. VTX00419 |
|  | 4YP04I95Y7_SS1 | MG321524 | *Glomus* sp pNTX E |
|  | 4YP04IA3LB_SS1 | MG321525 | *Glomus* sp. VTX00309 |
|  | 4YP04JG937_SS1 | MG321527 | *Glomus* sp. pNTX B |
|  | 4YP04H9ZQ1_SS1 | MG321528 | *Glomus* sp. pNTX L |
|  | 4YP04JH5JL_SS1 | MG321529 | *Glomus* sp. pNTX D |
|  | 4YP04IK9BE_SS1 | MG321530 | *Glomus* sp. VTX00419 |
|  | 4YP04IKWU3_SS1 | MG321531 | *Glomus* sp. VTX00151 |
|  | 4YP04JJNF5_SS1 | MG321532 | *Glomus* sp. VTX00419 |
|  | 4YP04I2LPU_SS1 | MG321533 | *Glomus* sp. pNTX G |
|  | 4YP04IL4N8_SS1 | MG321534 | *Glomus* sp. pNTX B |
|  | 4YP04JU5CY_SCS1 | MG321540 | *Glomus* sp. pNTX J |
|  | 4YP04IWR9Z_SCS1 | MG321542 | *Glomus* sp. VTX00151 |
|  | 4YP04JZGAX_SCS1 | MG321543 | *Glomus* sp. VTX00419 |
|  | YP04IOUW7_SCS1 | MG321545 | *Glomus* sp. VTX00098 |
|  | 4YP04JE2V8_SCS1 | MG321546 | *Glomus* sp. VTX00098 |
|  | 4YP04JASKM_SCS1 | MG321547 | *Glomus* sp. VTX00151 |
|  | 4YP04JFIIZ_SCS1 | MG321549 | *Glomus* sp. VTX00151 |
|  | 4YP04ITHCZ_SCS1 | MG321550 | *Glomus* sp. pNTX D |
|  | 4YP04JMQKY_SCS3 | MG321555 | *Glomus* sp. VTX00342 |
|  | 4YP04IVIMW_SCS3 | MG321556 | *Glomus* sp. pNTX M |
| **Non-assigned** | 4YP04IEEE0_SP1 | MG321432 | Glomeromycota sp. pNTX C |
|  | 4YP04I2XZV_SP2 | MG321457 | Glomeromycota sp. pNTX K |
|  | 4YP04I3GDA_SP2 | MG321459 | Glomeromycota sp. pNTX C |
|  | 4YP04I4LEN_SP3 | MG321472 | Glomeromycota sp. pNTX A |
|  | 4YP04IIHZP_SP3 | MG321486 | Glomeromycota sp. pNTX A |
|  | 4YP04JJQDS_SS1 | MG321526 | Glomeromycota sp. pNTX F |
|  | 4YP04JV0Q3_SCS1 | MG321539 | Glomeromycota sp. pNTX F |
|  | 4YP04INGAM_SCS1 | MG321541 | Glomeromycota sp. pNTX F |
|  | 4YP04IKJQV_SCS1 | MG321544 | Glomeromycota sp. pNTX C |
|  | 4YP04IZF3K_SCS1 | MG321548 | Glomeromycota sp. pNTX F |
