# Supplementary material for: Arbuscular mycorrhizal fungi in soil, roots and rhizosphere of Medicago truncatula: diversity and heterogeneity under semi-arid conditions
Source: PeerJ. 2019 Mar 1;7:e6401. doi: 10.7717/peerj.6401 (PMC6398376; doi:10.7717/peerj.6401)
Supplement: Table S2 — Number (#) and percentage (%) of OTUs from each specified group of organisms, detected in roots and rhizosphere (Rhiz) soils of M. truncatula and in bulk soils, from the studied sites. *- Includes representatives of Eukaryota (Metazoa, Viridiplant, Ciliophora, Alveolata) and Prokaryota (Bacteria). [file peerj-07-6401-s002.docx]

|  | **Site 1** | | | | | | **Site 2** | | | | | | **Site 3** | | | | | | **Site 4** | | | | | | **All sites** | | | | | |
| --- | --- | --- | --- | --- | --- | --- | --- | --- | --- | --- | --- | --- | --- | --- | --- | --- | --- | --- | --- | --- | --- | --- | --- | --- | --- | --- | --- | --- | --- | --- |
|  | Roots | | Rhiz. soil | | Bulk soil | | Roots | | Rhiz. Soil | | Bulk soil | | Roots | | Rhiz. soil | | Bulk soil | | Roots | | Rhiz. soil | | Bulk soil | | Roots | | Rhiz. Soil | | Bulk soil | |
| Taxon | OTU Distribution | | | | | | | | | | | | | | | | | | | | | | | | | | | | | |
|  | # | % | # | % | # | % | # | % | # | % | # | % | # | % | # | % | # | % | # | % | # | % | # | % | # | % | # | % | # | % |
| Ascomycota | 84 | 42 | 243 | 74 | 173 | 69 | 79 | 40 | 207 | 64 | 140 | 66 | 62 | 26 | 69 | 66 | 112 | 66 | 51 | 34 | 24 | 34 | 18 | 23 | 276 | 35 | 543 | 66 | 443 | 63 |
| Basidiomycota | 0 | 0 | 3 | 0.9 | 4 | 1.6 | 2 | 1 | 7 | 2.2 | 4 | 1.9 | 1 | 0.4 | 3 | 2.9 | 8 | 4.7 | 0 | 0 | 5 | 7.2 | 2 | 2.6 | 3 | 0.4 | 18 | 2.2 | 18 | 2.5 |
| Chytridiomycota | 0 | 0 | 0 | 0 | 0 | 0 | 0 | 0 | 7 | 2.2 | 2 | 0.9 | 0 | 0 | 0 | 0 | 0 | 0 | 0 | 0 | 0 | 0 | 0 | 0 | 0 | 0 | 7 | 0.8 | 2 | 0.3 |
| Early Diverging Fungal Lineages | 1 | 0 | 4 | 1.2 | 0 | 0 | 0 | 0 | 3 | 0.9 | 0 | 0 | 0 | 0 | 1 | 1 | 0 | 0 | 0 | 0 | 1 | 1.4 | 0 | 0 | 1 | 0.1 | 8 | 1 | 0 | 0 |
| **Glomeromycota** | 20 | **10.** | **13** | **3.9** | **12** | **4.8** | **27** | **14** | **0** | **0** | **4** | **1.9** | **48** | **20** | **0** | **0** | **2** | **1.2** | **12** | **8** | **0** | **0** | **0** | **0** | **107** | **14** | **13** | **1.6** | **18** | **2.5** |
| Other organisms* | 65 | 33 | 11 | 3.3 | 27 | 11 | 69 | 35 | 18 | 5.5 | 15 | 7.1 | 92 | 38 | 8 | 7.7 | 16 | 9.4 | 66 | 44 | 12 | 17 | 23 | 30 | 292 | 37 | 49 | 5.9 | 81 | 11 |
| Unidentified Eukaryota | 13 | 6.5 | 26 | 7.9 | 13 | 5.2 | 11 | 5.6 | 30 | 9.2 | 29 | 14 | 23 | 9.5 | 12 | 12 | 16 | 9.4 | 13 | 8.7 | 24 | 34 | 29 | 38 | 60 | 7.6 | 92 | 11 | 87 | 12 |
| Unidentified Fungi | 17 | 9 | 28 | 8.5 | 20 | 8 | 8 | 4.1 | 52 | 16 | 18 | 8.5 | 15 | 6.2 | 11 | 11 | 15 | 8.8 | 8 | 5.3 | 4 | 5.7 | 5 | 6.5 | 48 | 6.1 | 96 | 12 | 58 | 8.2 |
| **Total** | **200** |  | **328** |  | **249** |  | **196** |  | **324** |  | **212** |  | **241** |  | **104** |  | **169** |  | **150** |  | **70** |  | **77** |  | **787** |  | **826** |  | **707** |  |
