# Supplementary material for: Arbuscular mycorrhizal fungi in soil, roots and rhizosphere of Medicago truncatula: diversity and heterogeneity under semi-arid conditions
Source: PeerJ. 2019 Mar 1;7:e6401. doi: 10.7717/peerj.6401 (PMC6398376; doi:10.7717/peerj.6401)
Supplement: Table S3 — Virtual taxa (VTX) from the MaarjAM database with correspondence to the AMF OTUs identified in the DNA samples from M. truncatula roots and rhizosphere soils and from the bulk soils subjected to 454 pyrosequencing directed to the SSU rDNA region. [file peerj-07-6401-s003.docx]

| VTX code | Maarj*AM* database (N) | This study (n) | n/N (%) | Sites of Occurence | Species attributed in the Maarj*AM* database and in this study (*) | | | | | | |
| --- | --- | --- | --- | --- | --- | --- | --- | --- | --- | --- | --- |
| *Diversispora* sp. VTX00355 | 1 | 2 | 200 | 1, 4 |  | | | | | | |
| *Glomus* sp. VTX00307 | 3 | 1 | 33 | 3 |  | | | | | | |
| *Glomus* sp. VTX00331 | 7 | 2 | 29 | 3 |  | | | | | | |
| *Glomus* sp. VTX00098 | 10 | 2 | 20 | 1 |  | | | | | | |
| *Diversispora* sp. VTX00377 | 5 | 1 | 20 | 4 |  | | | | | | |
| *Glomus* sp. VTX00265 | 18 | 3 | 17 | 3 | ***G. coronatum**** *G. constrictum* *G. mosseae* | | | | | | |
| *Glomus* sp. VTX00151 | 46 | 6 | 13 | 1, 2 |  | | | | | | |
| *Glomus* sp. VTX00311 | 8 | 1 | 13 | 1 |  | | | | | | |
| *Diversispora* sp. VTX00380 | 40 | 3 | 8 | 3 |  | | | | | | |
| *Claroideoglomus* sp. VTX00357 | 14 | 1 | 7 | 4 |  | | | | | | |
| *Glomus* sp. VTX00419 | 97 | 5 | 5 | 1, 3 |  | | | | | | |
| *Glomus* sp. VTX00067 | 200 | 10 | 5 | 3, 4 | *G. mosseae G. coronatum* | | | | | | |
| *Diversispora* sp. VTX00054 | 119 | 5 | 4 | 3 | *Otospora barae D. celata D. aurantia Entrophospora nevadensis* | | | | | | |
| *Glomus* sp. VTX00108 | 176 | 6 | 3 | All |  | | | | | | |
| *Glomus* sp. VTX00309 | 38 | 1 | 3 | 1 |  | | | | | | |
| *Claroideoglomus* sp. VTX00193 | 551 | 14 | 3 | All | *C. claroideum C. lamellosum C. luteum C. infrequens C. etunicatum C. viscosum* | | | | | | |
| *Glomus* sp. VTX00105 | 255 | 6 | 2 | All | *G. intraradices* | | | | | | |
| *Glomus* sp. VTX00114 | 277 | 6 | 2 | All | *G. intraradices G. irregulare* | | | | | | |
| *Glomus* sp. VTX00065 | 94 | 2 | 2 | 3 | *G. geosporum G. fragilistratum G. verruculosum G. caledonium* | | | | | | |
| *Glomus* sp. VTX00280 | 146 | 3 | 2 | 1, 2, 3 |  | | | | | | |
| *Glomus* sp. VTX00387 | 51 | 1 | 2 | 1 |  | | | | | | |
| *Scutellospora* sp. VTX00049 | 122 | 2 | 2 | 3 | ***S. dipurpurescens**** *S. aurigloba S. calospora* | | | | | | |
| *Glomus* sp. VTX00115 | 554 | 9 | 2 | All | *G. intraradices G. irregulare G. vesiculiferum* | | | | | | |
| *Glomus* sp. VTX00342 | 281 | 4 | 1 | 2, 4 |  | | | | | | |
| *Glomus* sp. VTX00256 | 89 | 1 | 1 | 1 |  | | | | | | |
| *Glomus* sp. VTX00113 | 807 | 8 | 1 | 1, 2, 3 | *G. intraradices* | | | | | | |
| *Glomus* sp. VTX00295 | 123 | 1 | 1 | 3 |  | | | | | | |
| *Glomus* sp. VTX00177 | 146 | 1 | 1 | 3 |  | | | | | | |
| *Glomus* sp. VTX00092 | 325 | 2 | 1 | 3, 4 |  | | | | | | |
| *Glomus* sp. VTX00156 | 190 | 1 | 1 | 2 |  | | | | | | |
| *Glomus* sp. VTX00199 | 405 | 2 | 0 | 3 | *G. hoi G. macrocarpum* | | | | | | |
| *Glomus* sp. VTX00166 | 545 | 1 | 0 | 2 |  | | | | | | |
| 32 VTX | 5,743 matches | 113 OTUs |  |  |  |  |  |  |  |  |  |
