# Supplementary material for: Arbuscular mycorrhizal fungi in soil, roots and rhizosphere of Medicago truncatula: diversity and heterogeneity under semi-arid conditions
Source: PeerJ. 2019 Mar 1;7:e6401. doi: 10.7717/peerj.6401 (PMC6398376; doi:10.7717/peerj.6401)
Supplement: Table S4 — Pairwise distances matrix generated to constitute putative new taxa (pNTX) that group together OTU sequences sharing distances ≤3%. [file peerj-07-6401-s004.docx]

| pNTX | OTU |  | **1** | **2** | **3** | **4** | **5** | **6** | **7** | **8** | **9** | **10** | **11** | **12** | **13** | **14** | **15** | **16** | **17** | **18** | **19** | **20** | **21** | **22** | **23** | **24** |
| --- | --- | --- | --- | --- | --- | --- | --- | --- | --- | --- | --- | --- | --- | --- | --- | --- | --- | --- | --- | --- | --- | --- | --- | --- | --- | --- |
| **pNTX C** | 4YP04IEEE0_SP1 | **1** |  |  |  |  |  |  |  |  |  |  |  |  |  |  |  |  |  |  |  |  |  |  |  |  |
| **pNTX A** | 4YP04I4LEN_SP3 | **2** | 0.08 |  |  |  |  |  |  |  |  |  |  |  |  |  |  |  |  |  |  |  |  |  |  |  |
| **pNTX P** | 4YP04IHMFP_SP1 | **3** | 0.04 | 0.08 |  |  |  |  |  |  |  |  |  |  |  |  |  |  |  |  |  |  |  |  |  |  |
| **pNTX A** | 4YP04IIHZP_SP3 | **4** | 0.09 | 0.03 | 0.06 |  |  |  |  |  |  |  |  |  |  |  |  |  |  |  |  |  |  |  |  |  |
| **pNTX O** | 4YP04I0HTO_SP3 | **5** | 1.66 | 1.64 | 1.68 | 1.64 |  |  |  |  |  |  |  |  |  |  |  |  |  |  |  |  |  |  |  |  |
| **pNTX K** | 4YP04I2XZV_SP2 | **6** | 0.12 | 0.1 | 0.09 | 0.08 | 1.61 |  |  |  |  |  |  |  |  |  |  |  |  |  |  |  |  |  |  |  |
| **pNTX E** | 4YP04IW882_SP3 | **7** | 0.09 | 0.06 | 0.06 | 0.05 | 1.62 | 0.07 |  |  |  |  |  |  |  |  |  |  |  |  |  |  |  |  |  |  |
| **pNTX I** | 4YP04JCB34_SP2 | **8** | 0.08 | 0.08 | 0.07 | 0.08 | 1.64 | 0.09 | 0.06 |  |  |  |  |  |  |  |  |  |  |  |  |  |  |  |  |  |
| **pNTX N** | 4YP04IU4ZQ_SP3 | **9** | 0.06 | 0.08 | 0.05 | 0.07 | 1.58 | 0.07 | 0.05 | 0.05 |  |  |  |  |  |  |  |  |  |  |  |  |  |  |  |  |
| **pNTX C** | 4YP04I3GDA_SP2 | **10** | 0.03 | 0.09 | 0.05 | 0.1 | 1.63 | 0.13 | 0.1 | 0.09 | 0.07 |  |  |  |  |  |  |  |  |  |  |  |  |  |  |  |
| **pNTX H** | 4YP04I0PYT_SP2 | **11** | 0.07 | 0.08 | 0.04 | 0.07 | 1.64 | 0.1 | 0.07 | 0.06 | 0.06 | 0.08 |  |  |  |  |  |  |  |  |  |  |  |  |  |  |
| **pNTX J** | 4YP04JU5CY_SCS1 | **12** | 0.14 | 0.1 | 0.13 | 0.11 | 1.72 | 0.1 | 0.1 | 0.13 | 0.11 | 0.13 | 0.13 |  |  |  |  |  |  |  |  |  |  |  |  |  |
| **pNTX M** | 4YP04IVIMW_SCS3 | **13** | 0.13 | 0.1 | 0.1 | 0.09 | 1.68 | 0.11 | 0.05 | 0.1 | 0.09 | 0.14 | 0.11 | 0.14 |  |  |  |  |  |  |  |  |  |  |  |  |
| **pNTX B** | 4YP04IL4N8_SS1 | **14** | 0.06 | 0.07 | 0.05 | 0.06 | 1.61 | 0.09 | 0.07 | 0.08 | 0.05 | 0.07 | 0.06 | 0.1 | 0.11 |  |  |  |  |  |  |  |  |  |  |  |
| **pNTX G** | 4YP04I2LPU_SS1 | **15** | 0.09 | 0.11 | 0.09 | 0.1 | 1.65 | 0.1 | 0.1 | 0.09 | 0.07 | 0.06 | 0.09 | 0.11 | 0.14 | 0.06 |  |  |  |  |  |  |  |  |  |  |
| **pNTX L** | 4YP04H9ZQ1_SS1 | **16** | 0.11 | 0.09 | 0.09 | 0.09 | 1.65 | 0.1 | 0.09 | 0.1 | 0.08 | 0.12 | 0.09 | 0.13 | 0.13 | 0.06 | 0.09 |  |  |  |  |  |  |  |  |  |
| **pNTX F** | 4YP04JJQDS_SS1 | **17** | 0.07 | 0.09 | 0.06 | 0.1 | 1.64 | 0.12 | 0.07 | 0.12 | 0.1 | 0.08 | 0.09 | 0.17 | 0.12 | 0.08 | 0.13 | 0.12 |  |  |  |  |  |  |  |  |
| **pNTX B** | 4YP04JG937_SS1 | **18** | 0.06 | 0.07 | 0.06 | 0.06 | 1.65 | 0.07 | 0.06 | 0.06 | 0.04 | 0.06 | 0.05 | 0.08 | 0.11 | 0.03 | 0.04 | 0.05 | 0.1 |  |  |  |  |  |  |  |
| **pNTX F** | 4YP04IZF3K_SCS1 | **19** | 0.09 | 0.07 | 0.08 | 0.07 | 1.65 | 0.07 | 0.07 | 0.07 | 0.06 | 0.1 | 0.08 | 0.07 | 0.11 | 0.06 | 0.07 | 0.08 | 0.13 | 0.04 |  |  |  |  |  |  |
| **pNTX C** | 4YP04IKJQV_SCS1 | **20** | 0.02 | 0.08 | 0.04 | 0.09 | 1.61 | 0.12 | 0.09 | 0.08 | 0.06 | 0.03 | 0.07 | 0.14 | 0.13 | 0.06 | 0.09 | 0.11 | 0.07 | 0.06 | 0.09 |  |  |  |  |  |
| **pNTX F** | 4YP04JV0Q3_SCS1 | **21** | 0.08 | 0.07 | 0.07 | 0.07 | 1.65 | 0.07 | 0.06 | 0.06 | 0.05 | 0.09 | 0.07 | 0.07 | 0.1 | 0.05 | 0.06 | 0.07 | 0.12 | 0.03 | 0.02 | 0.08 |  |  |  |  |
| **pNTX D** | 4YP04ITHCZ_SCS1 | **22** | 0.08 | 0.07 | 0.07 | 0.07 | 1.67 | 0.07 | 0.06 | 0.06 | 0.05 | 0.08 | 0.07 | 0.06 | 0.1 | 0.05 | 0.05 | 0.07 | 0.12 | 0.02 | 0.02 | 0.08 | 0.01 |  |  |  |
| **pNTX F** | 4YP04INGAM_SCS1 | **23** | 0.09 | 0.06 | 0.06 | 0.06 | 1.68 | 0.07 | 0.05 | 0.07 | 0.06 | 0.09 | 0.07 | 0.07 | 0.09 | 0.05 | 0.07 | 0.07 | 0.11 | 0.04 | 0.03 | 0.09 | 0.02 | 0.02 |  |  |
| **pNTX D** | 4YP04JH5JL_SS1 | **24** | 0.08 | 0.05 | 0.05 | 0.05 | 1.67 | 0.06 | 0.04 | 0.05 | 0.05 | 0.09 | 0.06 | 0.08 | 0.08 | 0.03 | 0.06 | 0.05 | 0.09 | 0.03 | 0.04 | 0.08 | 0.03 | 0.03 | 0.02 |  |
| **pNTX E** | 4YP04I95Y7_SS1 | **25** | 0.09 | 0.06 | 0.07 | 0.05 | 1.62 | 0.05 | 0.03 | 0.07 | 0.06 | 0.1 | 0.08 | 0.08 | 0.07 | 0.05 | 0.08 | 0.07 | 0.09 | 0.05 | 0.05 | 0.09 | 0.05 | 0.05 | 0.04 | 0.02 |
