# Supplementary material for: Arbuscular mycorrhizal fungi in soil, roots and rhizosphere of Medicago truncatula: diversity and heterogeneity under semi-arid conditions
Source: PeerJ. 2019 Mar 1;7:e6401. doi: 10.7717/peerj.6401 (PMC6398376; doi:10.7717/peerj.6401)
Supplement: Table S5 — AMF OTUs without significant identity (≥97%) with taxa from the Maar jAM database and subjected to pairwise distance evaluation for the establishment of putative new taxa (pNTX). Each pNTX is either a single OTU or OTUs sharing distances ≤0.03. [file peerj-07-6401-s005.docx]

| Sequences evaluated by pairwise distances | | | Identification by BLAST (GenBank) | | | |  |
| --- | --- | --- | --- | --- | --- | --- | --- |
| GB acession number | OTU | Size (nt) | GB acession number | Identity | Coverage | Identification | Putative New Taxon (pNTX) |
| MG321471 | 4YP04IW882_SP3 | 333 | KY979412 | 100 | 100 | Uncultured *Glomus* sp. | *Glomus* sp. E |
| MG321524 | 4YP04I95Y7_SS1 | 443 | HF559361 | 99 | 100 | Uncultured *Glomus* sp. | *Glomus* sp. E |
| MG321529 | 4YP04JH5JL_SS1 | 540 | HF559296 | 98 | 99 | Uncultured *Glomus* sp. | *Glomus* sp. D |
| MG321550 | 4YP04ITHCZ_SCS1 | 444 | FJ913030 | 97 | 100 | Uncultured *Glomus* sp. | *Glomus* sp. D |
| MG321548 | 4YP04IZF3K_SCS1 | 329 | KP227248 | 97 | 100 | Uncultured Glomeromycota sp. | Glomeromycota sp. F |
| MG321541 | 4YP04INGAM_SCS1 | 327 | AM909675 | 97 | 100 | Uncultured Glomeromycota sp. | Glomeromycota sp. F |
| MG321451 | 4YP04I0PYT_SP2 | 465 | AM909675 | 97 | 100 | Uncultured *Glomus* sp. | *Glomus* sp. H |
| MG321540 | 4YP04JU5CY_SCS1 | 434 | FJ913030 | 97 | 91 | Uncultured *Glomus* sp. | *Glomus* sp. J |
| MG321457 | 4YP04I2XZV_SP2 | 318 | JQ350769 | 97 | 100 | Uncultured Fungus sp. | Glomeromycota sp. K |
| MG321502 | 4YP04IU4ZQ_SP3 | 316 | FM876881 | 97 | 100 | Uncultured *Glomus* sp. | *Glomus* sp. N |
| MG321472 | 4YP04I4LEN_SP3 | 359 | KY979470 | 96 | 98 | Uncultured *Diversispora* sp. | Glomeromycota sp. A |
| MG321527 | 4YP04JG937_SS1 | 437 | HF559361 | 96 | 100 | Uncultured *Glomus* sp. | *Glomus* sp. B |
| MG321526 | 4YP04JJQDS_SS1 | 382 | KP227071 | 96 | 99 | Uncultured Glomeromycota sp. | Glomeromycota sp. F |
| MG321539 | 4YP04JV0Q3_SCS1 | 328 | KP227110 | 96 | 100 | Uncultured Glomeromycota sp. | Glomeromycota sp. F |
| MG321447 | 4YP04JCB34_SP2 | 442 | AM909675 | 96 | 100 | Uncultured *Glomus* sp. | *Glomus* sp. I |
| MG321556 | 4YP04IVIMW_SCS3 | 330 | MG835549 | 96 | 100 | Uncultured *Glomus* sp. | *Glomus* sp. M |
| MG321425 | 4YP04IHMFP_SP1 | 536 | FN429383 | 96 | 100 | Uncultured *Glomus* sp. | *Glomus* sp. P |
| MG321534 | 4YP04IL4N8_SS1 | 330 | AM909675 | 95 | 100 | Uncultured *Glomus* sp. | *Glomus* sp. B |
| MG321432 | 4YP04IEEE0_SP1 | 465 | KF049916 | 95 | 100 | Uncultured Glomeromycota sp. | Glomeromycota sp. C |
| MG321459 | 4YP04I3GDA_SP2 | 462 | KP227251 | 95 | 99 | Uncultured Glomeromycota sp. | Glomeromycota sp. C |
| MG321544 | 4YP04IKJQV_SCS1 | 544 | KF186423 | 95 | 99 | Uncultured Glomeromycota sp. | Glomeromycota sp. C |
| MG321533 | 4YP04I2LPU_SS1 | 469 | HF559361 | 95 | 100 | Uncultured Glomus sp. | *Glomus* sp. G |
| MG321473 | 4YP04I0HTO_SP3 | 462 | GU353707 | 95 | 100 | Uncultured Glomus sp. | *Glomus* sp. O |
| MG321528 | 4YP04H9ZQ1_SS1 | 328 | AM909675 | 94 | 100 | Uncultured Glomus sp. | *Glomus* sp. L |
| MG321486 | 4YP04IIHZP_SP3 | 467 | JN009497 | 93 | 100 | Uncultured Glomeromycota sp. | Glomeromycota sp. A |
